# Regulatory Effects of *Codonopsis pilosula* Alkali-Extracted Polysaccharide Induced Intestinal *Lactobacillus* Enrichment on Peripheral Blood Proteomics in Tumor-Bearing Mice

**DOI:** 10.3390/microorganisms13081750

**Published:** 2025-07-26

**Authors:** Yuting Fan, Chenqi Yang, Yiran Zhao, Xiao Han, Hongfei Ji, Zhuohao Ren, Wenjie Ding, Haiyu Ji

**Affiliations:** 1 School of Life Sciences, Yantai University, Yantai 264005, China; fyting7766@163.com (Y.F.); ycq1327@163.com (C.Y.); zyran6789@163.com (Y.Z.); hanxiao7721@163.com (X.H.); hongfei10052024@163.com (H.J.); rzhao6789@163.com (Z.R.); 2 College of Food Science and Engineering, Tianjin University of Science and Technology, Tianjin 300457, China; dingwenjie1120@163.com

**Keywords:** *C. pilosula* alkali-extracted polysaccharide, gut microbiota compositions, antitumor immunity, label-free proteomics

## Abstract

*Codonopsis pilosula* polysaccharides have demonstrated multiple biological activities including immune regulation, antitumor, and antioxidant properties. The rapid development and integrated application of multi-omics can facilitate the unraveling of the complex network of immune system regulation. In this study, *C. pilosula* alkali-extracted polysaccharide (CPAP) were prepared, and their effects on gut microbiota compositions, metabolic pathways, and protein expressions in peripheral blood and solid tumors in mice were further evaluated. The 16S rDNA sequencing results showed that CPAP could effectively promote the enrichment of intestinal *Lactobacillus* in tumor-bearing mice. In addition, it could be inferred from peripheral blood and solid tumor proteomics results that CPAP might activate T cell-mediated antitumor immune functions by regulating purine metabolism and alleviate tumor-caused inflammation by promoting neutrophil degranulation, finally inducing apoptosis in tumor cells by increasing oxidative stress. These results will provide a theoretical foundation and data support for the further development of CPAP as dietary adjuvants targeting immune deficiency-related diseases.

## 1. Introduction

*Codonopsis pilosula* is a plant with both medicinal and edible properties and has exhibited the functions of invigorating the spleen and nourishing the lungs. *C. pilosula* polysaccharides have demonstrated significant immunomodulatory, antioxidant, and antitumor activities [1], and the therapeutic effects on immunodeficient hosts are mainly achieved through the modulation of gut microbiota compositions and metabolism [2]. In addition, *C. pilosula* polysaccharides can alleviate sterigmatocystin-induced intestinal dysbiosis by modulating the diversity of the gut microbiota [3] and significantly restore intestinal mucosal immune function and attenuate inflammatory responses in cyclophosphamide-induced immunosuppressed mice [4]. In our previous study, a *C. pilosula* glucofructan with low molecular weights was prepared using neutral water as the solvent and effectively enhanced humoral and cellular immunity in tumor-bearing mice with an inhibitory rate of 45.37% (100 mg/kg) [5]. However, the alkali-extracted polysaccharides presented a higher dietary fiber content and exhibited better regulatory effects on intestinal probiotics [6]. In addition, the preliminary experimental results (Supplementary Figure S1) also demonstrated that *C. pilosula* alkali-extracted polysaccharide (CPAP) presented superior antitumor and immunomodulatory activities.

Tumors are new organisms formed by the abnormal proliferation of body cells under the influence of tumorigenic factors, which have become a public health challenge involving multiple dimensions such as physiology, psychology, and society [7]. The development of tumors can impair immune responses and disrupt the body’s homeostasis [8]. The tumor microenvironment (TME), a complex ecosystem in which tumor cells interact with surrounding stromal components, immune cells, and microbiota, plays pivotal roles in tumor initiation, progression, and metastasis [9]. Persistent inflammatory responses in the TME can exacerbate immune dysregulation and facilitate the rapid growth and immune evasion of tumor cells [10].

As the largest microbial ecosystem in the human body, gut microbiota are composed of approximately 3 × 10^13^ bacteria [11], which hold great significance in preventing and managing metabolic disorders. Dietary fibers (including polysaccharides) can affect the gut microbiota metabolism and thereby reshape microbial compositions and regulate host physiological functions [12]. In addition, gut microbiota demonstrate crucial roles in the host’s immune function processes and the development of tumor cells [13]. Gut dysbiosis can suppress immune responses and induce inflammatory status through microbial metabolites, thereby promoting immunosuppressive ecosystem construction in the TME [14]. Therefore, the regulation of the gut microbiota composition and metabolites to restore antitumor immune functions has become an important strategy for tumor immunotherapy.

In this study, *C. pilosula* alkali-extracted polysaccharide (CPAP) were prepared, and the immunomodulatory effects of CPAP on tumor-bearing mice were investigated using multi-omics technologies, particularly focusing on the systematic regulation of gut microbiota compositions, metabolites expressions, and proteins distributions in peripheral blood and solid tumors. These findings will provide novel experimental evidence supporting the immunomodulatory mechanisms of CPAP via gut microbiota metabolism and a data-based foundation for further development in the field of functional foods.

## 2. Materials and Methods

### 2.1. Preparation of CPAP

The dried *C. pilosula* roots were pulverized and immersed in alkaline electrolytic aqueous solution (pH = 12, −1100 mV) under a liquid-to-solid ratio of 30 mL/g, extraction temperature of 80 °C, and extraction time of 2 h, and then the extracts were collected and concentrated under vacuum condition. The concentrated solution was treated with the addition of 3 times the volume of absolute ethanol, the precipitates were redissolved in distilled water, and CPAP were obtained after membrane filtration (with a molecular weight cut off of 1000 Da) and vacuum freeze drying.

### 2.2. Experimental Animals and Tumor Model Establishment

The forty female Kunming mice (8 weeks old, Specified Pathogen-Free grade, 25 ± 2 g) were purchased from Jinan Pengyue Experimental Animal Breeding Co., Ltd. (Jinan, China) with the production certificate No. of SCXK(Lu)2022-0006. All animal experiments were conducted with the approval of the Ethics Committee of Tianjin University of Science & Technology (approval number 2024045, granted on 16 October 2024), which complied with the U.K. Animals (Scientific Procedures) Act of 1986 and associated guidelines. All mice were housed in an animal facility under controlled environmental conditions including a constant room temperature of 22 ± 2 °C, relative humidity of 50 ± 5%, and a 12 h light/dark cycle.

After acclimatization, the mice were randomly divided into 4 groups (10 mice per group): a blank group, a model group, and CPAP groups (50 and 100 mg/kg). The CPAP groups received daily oral gavage of CPAP solution for 7 days, while the blank and the model groups were administered equivalent volume of physiological saline. On day 8, all mice were subcutaneously inoculated with S180 sarcoma cells (2 × 10^6^ cells/mouse) into the right axillary region except for the blank group, and the gavage administration was continued for another 14 days; finally, the experiment was terminated on day 22 [15].

The solid tumors of these groups were excised and weighed, and the tumor inhibitory rate (%) was determined as [1 − (tumor weight of treatment group/tumor weight of the model group)] × 100 [16]; the results showed that CPAP could effectively suppress tumor growth in mice with inhibitory rates of 43.75% (50 mg/kg) and 59.52% (100 mg/kg), indicating superior antitumor potential in a dose-dependent manner.

### 2.3. Determination of 16S rDNA Amplicon 

The gut microbiota compositions were analyzed by using 16S rDNA gene sequencing technology on mouse fecal samples. On day 22, the fecal samples were collected, and the DNA was extracted using the QIAamp DNA Stool Mini Kit (QIAGEN NV Hilden, Germany); V3-V4 hypervariable regions were amplified using the primer pair 338F (5′-ACTCCTACGGGAGGCAGCAG-3′) and 806R (5′-GGACTACHVGGGTWTCTAAT-3′) on a polymerase chain reaction (PCR) system. The PCR products were purified and subsequently sequenced on the Illumina MiSeq platform and analyzed with the QIIME2 platform [17]. After removing the barcode and primer sequences (reads that received an average quality score of less than 20 were trimmed using Trimmomatic software (version of 0.38) with an alternative sliding window, whereby those shorter than 50 bp were discarded), FLASH (V1.2.7, http://ccb.jhu.edu/software/FLASH/) was used to assemble the reads of each sample, and the assembled sequences underwent strict filtering processing to obtain high-quality Tags data. Subsequently, the effective tags were obtained through comparison and detection in https://github.com/torognes/vsearch/ (accessed on 10 December 2024) with Operational Taxonomic Unit (OTU) clustering according to the 97% similarity cut-off. Finally, gut microbiota composition analysis was conducted through Sequence Read Archive (http://www.ncbi.nlm.nih.gov/Traces/sra, accessed on 10 December 2024) database comparison. Finally, the top 10 gut bacteria in terms of relative abundance (selected from the overall abundance across all experimental groups combined) at family and genus levels were screened and discussed.

### 2.4. Intestinal Metabolic Product Determination

Untargeted metabolomics was utilized to analyze the metabolites in tumor-bearing mice [16]. Fecal samples from both the model and CPAP groups were gathered on day 22, freeze-dried using liquid nitrogen, and subsequently pulverized into a fine powder. Next, 100 mg samples were precisely weighed and combined with 500 μL methanol solution (80%). After a 5 min ice bath, the samples were centrifuged (15,000× *g*, 20 min) at 4 °C, and the supernatants were collected using liquid chromatography–mass spectrometry (LC-MS), which was performed on a Dionex Ultimate 3000 system fitted with a Thermo Syncronis C18 column (2.1 mm × 100 mm, Waltham, MA, USA), and analyzed through Trace Finder software (version 3.2.0). The gradient elution conditions were set as follows: 0~1 min, 95% A; 1~5 min, decreasing from 95% to 40% A; 5~8 min, decreasing from 40% to 0% A; 8~11 min, maintaining 0% A; 11~14 min, increasing from 0% to 40% A; 14~15 min, increasing from 40% to 95% A; 15~18 min, maintaining 95% A. Solvent A comprised water with 0.1% formic acid and 2 mM ammonium formate, while solvent B was acetonitrile.

### 2.5. Differential Protein Determination

Label-free technology was employed for determining the differential proteins in mouse sera, leukocytes, and solid tumor cells between the model and CPAP groups [18]. On day 22, the peripheral blood was obtained from each group by orbital blood collection and divided into 2 tubes. One was placed at 4 °C for 2 h and centrifuged (3000× *g*, 10 min), then the supernatant was collected (serum). The other was immediately added with twice the volume of red blood cell lysis buffer for 20 min. After the removal of lysed erythrocytes by centrifugation (3000× *g*, 10 min), the sediment was collected (leukocyte). In addition, the solid tumor could be directly excised from the armpits of mice.

These samples were cryogenically pulverized, and then the protein lysate was added, followed by low-temperature ultrasonication. After denaturation at 95 °C for 8 min, the pre-cooled 80% acetone was added to precipitate the proteins, which were further digested using trypsin. Finally, the samples were analyzed through LC-MS (Thermo Fisher Scientific, Waltham, MA, USA) equipped with a C18 column in conjunction with timsTOF Pro2 (Bruker Daltonics, Billerica, MA, USA). The differentially expressed proteins between the model and CPAP groups were identified and subjected to functional annotation and enrichment analysis from https://cn.string-db.org (accessed on 30 December 2024), including Gene Ontology (GO), KEGG pathway, Reactome pathway, and Monarch database analyses.

### 2.6. Data Analysis

All experimental data were acquired from at least three parallel experiments and analyzed using SPSS statistical software (version 20.0). Comparisons among multiple groups were performed using one-way analysis of variance (ANOVA) with Tukey’s test, and a *p* value < 0.05 was considered statistically significant. In addition, principal component analysis (PCA) was employed for gut microbiota compositions analysis, partial least squares discriminant analysis (PLS-DA) was applied for the inter-group differential metabolite analysis, and the False Discovery Rate (FDR < 0.05) was used for the functional annotation and enrichment analysis in proteomics.

## 3. Results

### 3.1. Gut Microbiota Composition Results

**Figure 1:**
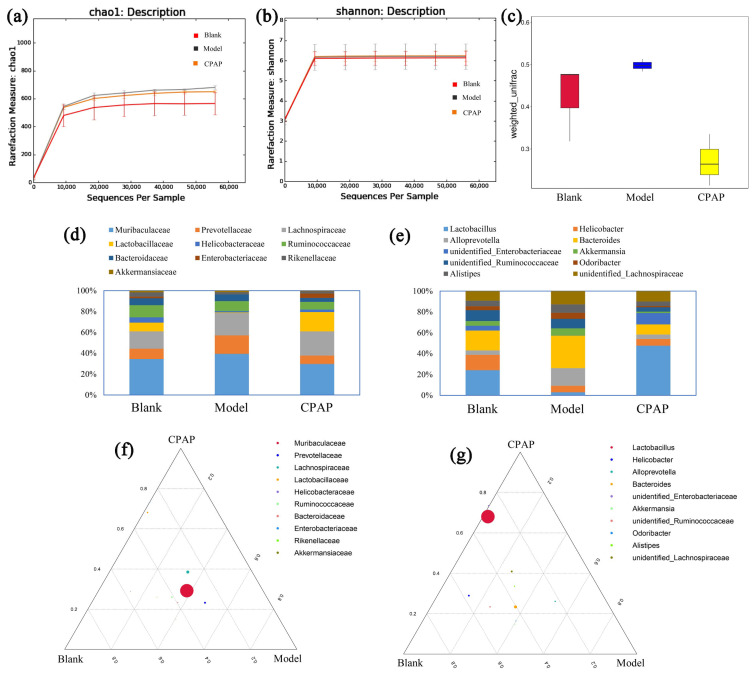
The impacts of CPAP on gut microbiota compositions. (**a**) chao1 description; (**b**) Shannon description; (**c**) weighted unifrac; (**d**) proportions at family level; (**e**) proportions at genus level; (**f**) ternary-phase diagram at family level; (**g**) ternary-phase diagram at genus level.

The effects of CPAP on gut microbiota compositions were determined, and the results are shown in Figure 1. Figure 1a,b present the α-diversity results including Chao1 and Shannon curves. As shown, as the sequences gradually increased, the rarefaction curves of the gut microbiota in each group displayed a rapid rise followed by gradual flattening, suggesting that the sequencing depth of these samples basically covered the main species compositions of the gut microbiota, and the diversity and abundances among different groups were at a similar level [19]. Figure 1c demonstrates the β-diversity results of these groups. The weighted unifrac among the blank, model, and CPAP groups exhibited obvious differences, indicating that CPAP did not affect the total species number of gut microbiota while effectively regulating the relative abundances of some bacteria [20].

As shown in Figure 1d,f, compared with the model group at the family level, the abundances of *Lactobacillaceae* were significantly improved (*p* < 0.05) in the blank and CPAP groups. *Lactobacillaceae* plays crucial roles in maintaining gut homeostasis by producing lactic acid and other metabolites to lower the intestinal pH, which can effectively enhance antitumor immunity, mitigate chronic inflammation, and alleviate tumor-induced microbial dysbiosis [21].

As presented in Figure 1e,g, compared with the model group at the genus level, the *Lactobacillus* contents in the blank and CPAP groups were remarkably increased (*p* < 0.05), while the *Alloprevotella* contents were significantly reduced (*p* < 0.05). *Lactobacillus* can effectively enhance immune responses via the immunogenic components (peptidoglycan and lipoteichoic acid) and relevant metabolites (short-chain fatty acids, bacteriocins, and exopolysaccharides) [22], suggesting that CPAP can help restore microbial balance, reduce inflammation, and support intestinal barrier function, which was consistent with previous results showing *Lactobacillus* enrichment induced by *Lactarius deliciosus* polysaccharide [23]. However, the high abundances of *Alloprevotella* may facilitate the malignant proliferation or immune escape of tumor cells in the model group [24].

Therefore, CPAP mainly improved the intestinal *Lactobacillus* contents while reducing the *Alloprevotella* contents at the genus level in tumor-bearing mice, which might be relevant to the enhanced antitumor immunity.

### 3.2. Analysis of Differential Metabolites Between the Model and CPAP Groups

**Figure 2:**
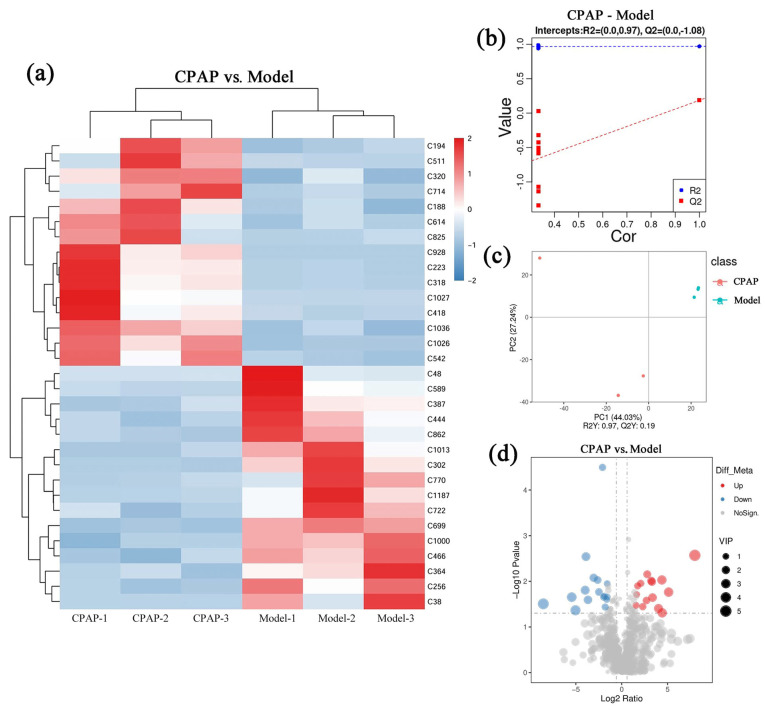
Impacts of CPAP on intestinal metabolism of tumor-bearing mice. (**a**) Differential metabolite cluster heat diagram; (**b**) PLS-DA plot; (**c**) PCA plot; (**d**) volcano diagram.

Untargeted metabolomics was employed to quantitatively and qualitatively analyze intestinal metabolites in the mice of the model and CPAP groups. There were 31 metabolites with significant differential expressions, of which 15 were upregulated and 16 were downregulated in the CPAP group compared with the model group. In Figure 2a, the color gradient from blue to red represents increasing metabolite concentrations, providing a visual representation of the differential expression patterns between the two groups. In addition, the specific information of these differential metabolites is provided in Supplementary Table S1. Figure 2b displays the results of partial least squares discriminant analysis (PLS-DA). The R^2^ value exceeds Q^2^ and approaches 1, indicating the high explanatory ability and good generalization ability of the model. The Q^2^ regression line shows a negative intercept on the *Y*-axis, further confirming the model’s good fit without overfitting [25]. Figure 2c presents the results of principal component analysis (PCA). PC1 and PC2 demonstrate variances of 44.03% and 27.24%, respectively, which suggests that the differences in principal components between groups were significant, indicating the holistic regulatory effects of CPAP on the intestinal metabolic network. Figure 2d shows a volcano plot visualizing the differential expressions of these metabolites between the CPAP and model groups, where red dots represent significantly upregulated metabolites, blue dots indicate significantly downregulated metabolites, and gray dots denote metabolites with no significant changes. Metabolites with a variable importance in projection (VIP) value greater than 1 were identified as key discriminators between the two groups. These results demonstrated that CPAP can significantly regulate the expressions of these intestinal metabolites, while the impacts on physiological functions still require further analysis.

### 3.3. Analysis of Highly Expressed Metabolites in the Model Group

**Figure 3:**
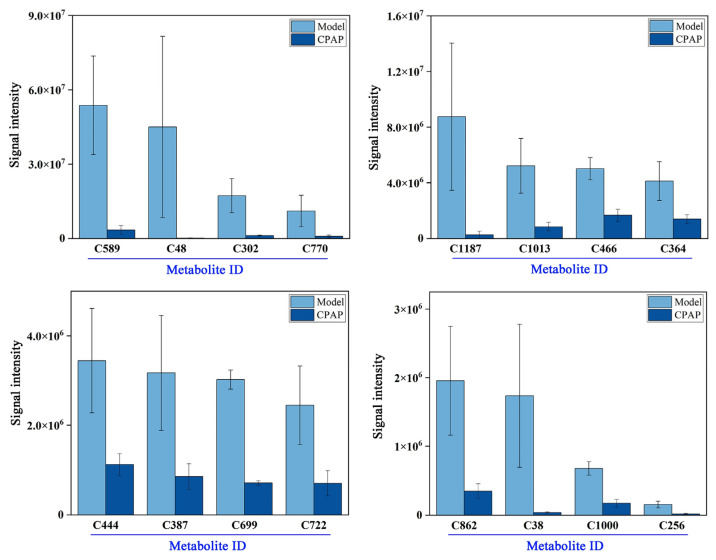
Histograms of highly expressed metabolites in the model group.

As illustrated in Figure 3, 16 metabolites were highly expressed in the model group compared with the CPAP group, and the compound information corresponding to the Meta ID is given in Supplementary Table S1.

LPC 14:0 (C589, lysophosphatidylcholine 14:0) is associated with tumor progression and recurrence and can be used as a biomarker for certain cancers [26]; elevated levels of LPC may promote the release of inflammatory cytokines, such as TNF-α and IL-6, which show a positive correlation with the malignant proliferation of cancer cells. The content of PC (15:0/16:0) (C770) in breast cancer cells is approximately 40% higher than that in normal breast epithelial cells, indicating that the membrane property of high rigidity is positively correlated with the invasiveness of cancer cells [27]. Dimethyl-2-(3-nitro-2-pyridyl)-malonate (C1187) may be associated with mitochondrial dysfunction mediated by succinate dehydrogenase in tumor cells [28]. Uridine (C1013), a critical component of cellular nucleic acids, plays crucial roles in protein and nucleic acid biosynthesis and energy production [29], influencing cell proliferation and repair. Cancer cells can universally utilize uridine as a nutrient and energy source. Therefore, the high expression of uridine in the model group may reflect enhanced uptake and utilization of uridine by cancer cells to support malignant proliferation. Ecgonine methyl ester (C466) can induce oxidative stress responses in tumor-bearing hosts, thereby triggering potential DNA damage risks [30], which may be related to the high oxidative characteristics in the TME. Carvone (C364) can mitigate oxidative stress-induced cellular damage by scavenging reactive oxygen species (ROS) [31], and the high expression in the model group may reflect the adaptive response to increased oxidative stress by upregulating carvone production. Dl-citrulline (C444) and citrulline (C387) are the key intermediates in the arginine metabolic pathway and have been proven to promote tumor growth [32]. Additionally, as a precursor to nitric oxide (NO) synthesis, the elevated expression of citrulline may also promote NO production, supporting tumor angiogenesis [33]. N-heptanoylhomoserine lactone (C722), a bacterial quorum-sensing molecule [34], exhibited the high expression in the model group, potentially reflecting gut microbiota dysbiosis and abnormal quorum sensing, further exacerbating intestinal microenvironment disturbances. In addition, the other highly expressed compounds are likely to be related to the promotion of tumor growth, meaning that these results provide data support for future research in this area.

In summary, the high expression of these metabolites in the model group indicated that the malignant proliferation of solid tumors could cause inflammatory response, an high oxidative stress environment, and the imbalance of gut microbiota in hosts.

### 3.4. Analysis of Highly Expressed Metabolites in the CPAP Group

**Figure 4:**
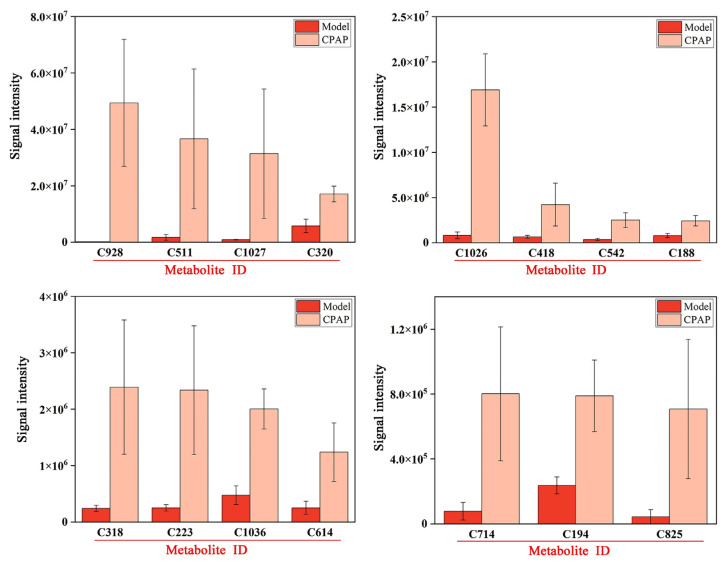
Histograms of highly expressed metabolites in the CPAP group.

The expressions of 15 specific metabolites were significantly improved in the CPAP group compared with the model group, and the results are shown in Figure 4.

RMK (C928) is typically downregulated in tumor-bearing hosts; the upregulation after CPAP treatment may be relevant to tumor growth inhibition through immunomodulatory activity [16]. Glycerophospho-N-palmitoylethanolamine (C511) is a metabolic precursor of palmitoylethanolamide (PEA). PEA is an endocannabinoid widely present in the liver, brain, and other mammalian tissues that has shown definite anti-inflammatory activity [34]. Therefore, CPAP could promote PEA biosynthesis and effectively inhibit inflammatory signaling pathways in tumor-bearing mice, thereby improving the gut microenvironment. Adenine (C320) serves as a signaling molecule that converts incoming signals into appropriate cellular responses, such as the activation of immune reactions against pathogens [35]. The elevated expressions in the CPAP group indicated the effective enhancement of antitumor immunity in tumor-bearing mice. Dehydrocholic acid (C418) and hyodeoxycholic acid (C542) were significantly upregulated in the CPAP group; they possess anti-inflammatory [36] and antitumor [37] properties as bile acid metabolites and also play crucial roles in maintaining the gut microbiota composition and metabolic activity [38]. In addition, (1,2,3,5,9,18)-1,2,3,19-tetrahydroxyurs-12-en-28-oic acid (C1026) and 18-glycyrrhetinic acid (C1036), as pentacyclic triterpenoids, have also been proven to exhibit anti-inflammatory and antitumor effects [39]. These results suggest that CPAP could alleviate the inflammatory responses caused by tumors and effectively improve the intestinal immune capacities. Actarit (C318), an effective immunomodulator, plays an important role in the intervention of autoimmune diseases [40]. The elevated expression in the CPAP group demonstrated that CPAP could effectively enhance antitumor immune activity. LPE 22:5 (C614) and PC (19:1/20:5) (C825), as phospholipid metabolites, were significantly upregulated in the CPAP group, and the upregulation may help restore the intestinal barrier and maintain epithelial homeostasis. LPE 22:5 (C614) and PC (19:1/20:5) (C825), as the phospholipid metabolites, were significantly upregulated in the CPAP group, which might contribute to the restoration of intestinal barrier function and the maintenance of intestinal cellular homeostasis by modulating specific signaling pathways associated with gut health [41]. 3-keto Fusidic acid (C194) is an antibiotic derivative that may regulate gut microbiota compositions by inhibiting the growth of harmful bacteria in the CPAP group [42].

In summary, these metabolites alterations indicated that CPAP presented strong antitumor, immunoregulation, anti-inflammatory effects in tumor-bearing mice after inducing *Lactobacillus* enrichment.

### 3.5. Analysis of Highly Expressed Proteins in Sera from the Model Group

**Figure 5:**
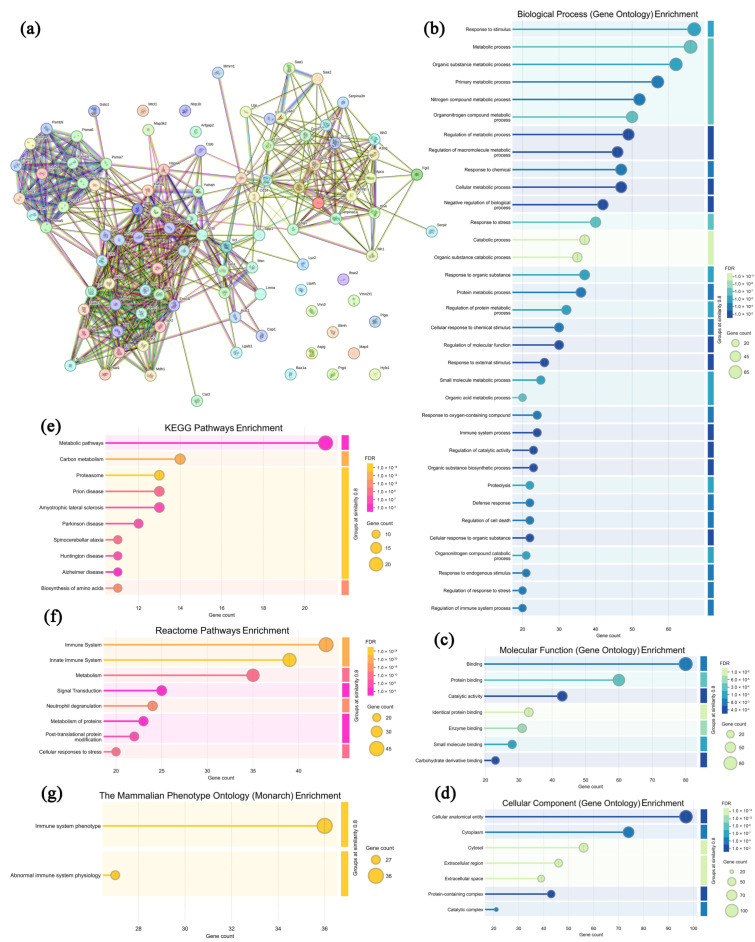
Association network of highly expressed serum proteins in the model group (**a**) and functional analysis ((**b**–**d**) GO annotations; (**e**–**g**) signaling pathways).

In mouse sera from the model group, 100 highly expressed proteins were selected and analyzed with the specific information presented in Supplementary Table S2, and the associated protein network (Figure 5a) and functional annotation analysis (Figure 5b–d, GO annotation; Figure 5e–g, pathway analysis) are illustrated in Figure 5. As shown in Figure 5a, most of the highly expressed proteins in the model group exhibit specific interactions, with three notable clusterings of proteins in the upper-left and lower-left quadrants. In Figure 5b–d, these proteins are primarily enriched in the 12 biological processes (response to stimulus, metabolic process, organic-substance metabolic process, primary metabolic process, nitrogen compound metabolic process, etc.), seven molecular functions (binding, protein binding, catalytic activity, identical protein binding, enzyme binding, etc.), and six cellular components (the cellular anatomical entity, cytoplasm, cytosol, extracellular region, extracellular space, protein-containing complex, and catalytic complex), which suggests that the cell proliferation in solid tumors occurred at a rapid rate with elevated metabolic activity [43]. As shown in Figure 5e–g, in the KEGG database, these proteins are enriched in 10 pathways, including metabolic pathways, carbon metabolism, proteasome, prion disease, and amyotrophic lateral sclerosis, which indicates that tumor cells enhance protein degradation to maintain intracellular protein homeostasis, supporting their rapid proliferation and survival [44]. In the Reactome database, these proteins were enriched in eight pathways, including the immune system, the innate immune system, metabolism, signal transduction, neutrophil degranulation, etc. Additionally, in the Monarch database, pathways including immune system phenotype and abnormal immune system physiology were enriched. Therefore, it can be inferred that the malignant proliferation of tumor cells leads to the activation of non-specific immunity and the occurrence of inflammatory responses in the body [45].

### 3.6. Analysis of Highly Expressed Proteins in Sera from the CPAP Group

**Figure 6:**
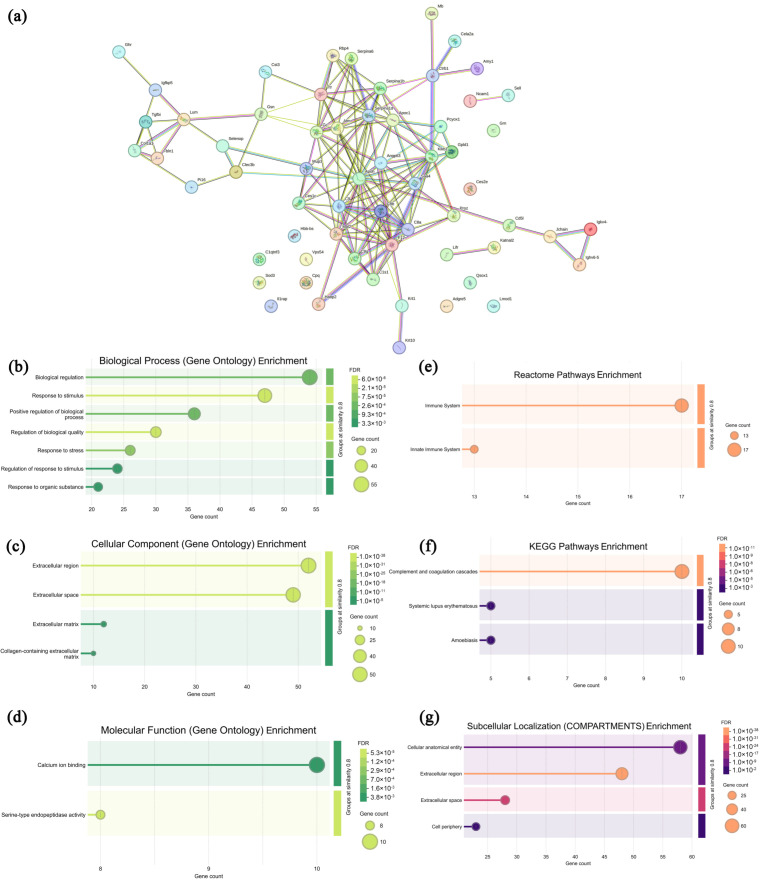
Association network of highly expressed serum proteins in the CPAP group (**a**) and functional analysis ((**b**–**d**) GO annotations; (**e**–**g**) signaling pathways).

In mouse sera from the CPAP group, 77 highly expressed proteins were selected and analyzed with the specific information presented in Supplementary Table S3, and the associated network Figure 6a and functional annotation analysis (Figure 6b–d, GO annotation; Figure 6e–g, pathway analysis) are presented in Figure 6. As shown in Figure 6a, most of the highly expressed proteins in the CPAP group exhibited specific interactions, suggesting their potential involvement in the regulation of certain signaling pathways, particularly the protein clusters concentrated in the central region. In Figure 6b–d, these highly expressed proteins were primarily enriched in seven biological processes (biological regulation, response to stimuli, the positive regulation of biological process, the regulation of biological quality, response to stress, etc.), four cellular components (the extracellular region, extracellular space, extracellular matrix, and collagen-containing extracellular matrix), and two molecular functions (calcium ion binding and serine-type endopeptidase activity), indicating that CPAP promoted apoptosis in tumor cells [46]. As shown in Figure 6e–g, in the Reactome database, these proteins are enriched in the immune system and innate immune system pathways, suggesting that CPAP intervention also enhances innate immunity [47]. In the KEGG database, the complement and coagulation cascades, systemic lupus erythematosus, and amoebiasis pathways are enriched, suggesting that CPAP may enhance complement component (C1q, C3a, C5a) activities to effectively eliminate tumor cells, suppressing tumor angiogenesis and metastasis [48,49]. In the COMPARTMENTS database, these proteins are enriched in subcellular localizations such as the cellular anatomical entity, extracellular region, extracellular space, and cell periphery, revealing the significant impacts of CPAP intervention in the regulation of extracellular matrix remodeling and intercellular communications [50]. Therefore, it may be deduced that CPAP intervention can improve immune system functions and eventually induce the apoptosis of tumor cells.

### 3.7. Analysis of Highly Expressed Proteins in Leukocytes from the Model Group

**Figure 7:**
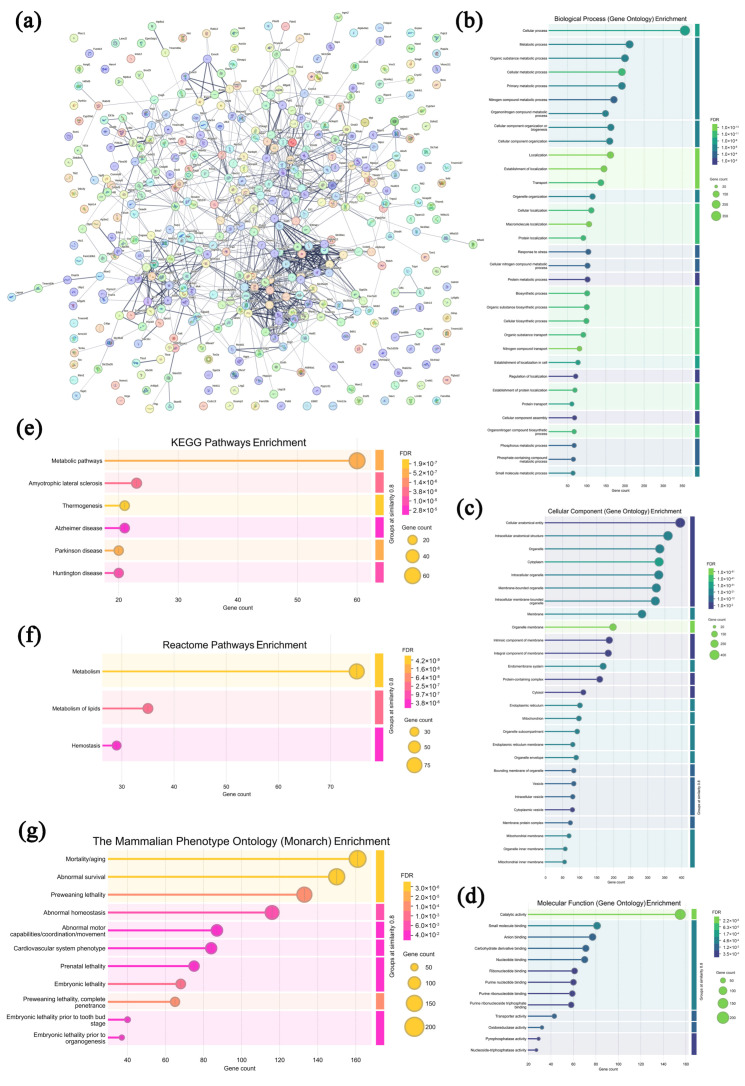
Association network of highly expressed leukocyte proteins in the model group (**a**) and functional analysis ((**b**–**d**) GO annotations; (**e**–**g**) signaling pathways).

In the leukocytes from the model group, 431 highly expressed proteins were screened and analyzed compared with the CPAP group, and the specific information is displayed in Supplementary Table S4. As shown in Figure 7a, most of the highly expressed proteins in the model group exhibited specific interactions, collectively revealing diverse biological functions and complex regulatory networks. In Figure 7b–d, these proteins are seen to be primarily enriched in 12 biological processes (the cellular process, metabolic process, organic substance metabolic process, cellular metabolic process, primary metabolic process, etc.), 14 cellular components (the cellular anatomical entity, intracellular anatomical structure, organelle, cytoplasm, intracellular organelle, etc.), and nine molecular functions (catalytic activity, small-molecule binding, anion binding, carbohydrate derivative binding, nucleotide binding, etc.), indicating that the uncontrolled growth of solid tumors affected the fundamental cellular activities and metabolic processes of leukocytes [51,52]. As shown in Figure 7e–g, in the KEGG database, these proteins are enriched in six pathways, including metabolic pathways, amyotrophic lateral sclerosis, thermogenesis, Alzheimer’s disease, Parkinson’s disease, and Huntington’s disease, suggesting that solid tumors could suppress the leukocyte activities [53]. In the Reactome database, 3 pathways were enriched, including metabolism, the metabolism of lipids, and hemostasis, while in the Monarch database, 11 pathways were enriched, including mortality/aging, abnormal survival, preweaning lethality, abnormal homeostasis, etc., demonstrating that tumor cell proliferation obviously affects the survival, development, and homeostasis of normal immune cells [54,55]. Therefore, it can be speculated that the malignant growth of solid tumors remarkably disrupts the development and homeostasis of leukocytes, thereby weakening the body’s immune capacities.

### 3.8. Analysis of Highly Expressed Proteins in Leukocytes from the CPAP Group

**Figure 8:**
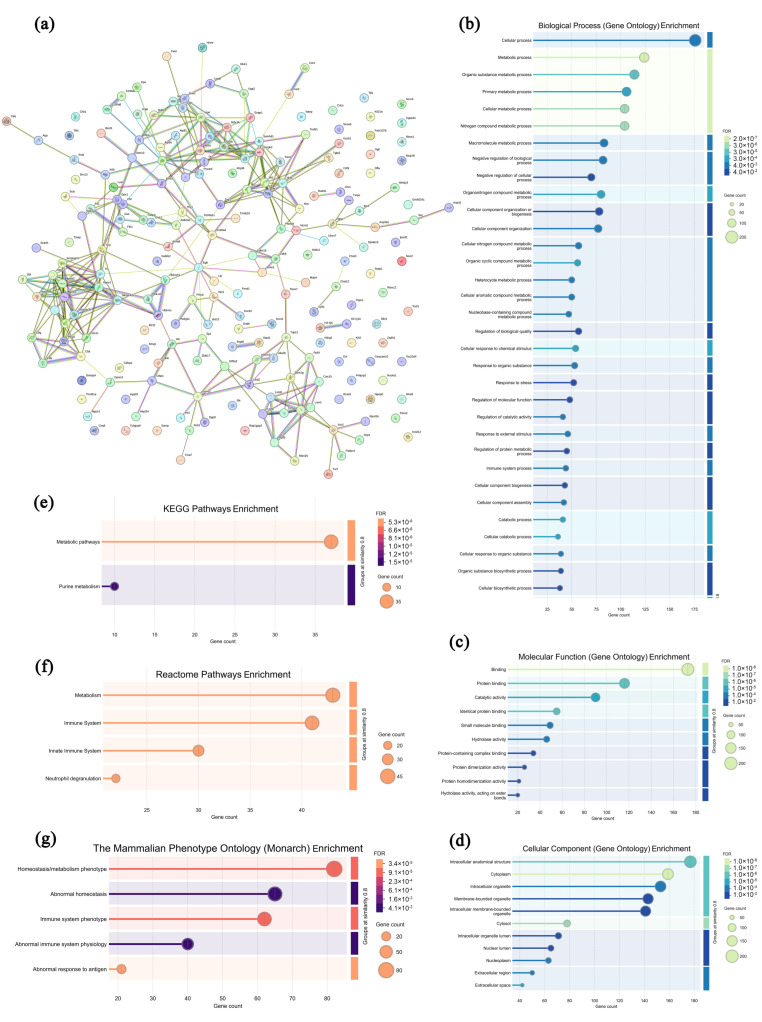
Association network of highly expressed leukocyte proteins in the CPAP group (**a**) and functional analysis ((**b**–**d**) GO annotations; (**e**–**g**) signaling pathways).

In the leukocytes from the CPAP group, 212 highly expressed proteins were screened and analyzed compared with the model group, and the specific information is shown in Supplementary Table S5. As presented in Figure 8a, most of the highly expressed proteins in the CPAP group exhibited specific interactions, suggesting that these proteins may synergistically regulate autoimmune responses and biological processes. In Figure 8b–d, these highly expressed proteins are primarily enriched in 12 biological processes (the cellular process, metabolic process, organic substance metabolic process, primary metabolic process, cellular metabolic process, etc.), 10 molecular functions (binding, protein binding, catalytic activity, identical protein binding, small molecule binding, etc.), and 11 cellular components (the intracellular anatomical structure, cytoplasm, intracellular organelle, membrane-bounded organelle, intracellular membrane-bounded organelle, etc.), indicating that CPAP could maintain the normal structure and functions of leukocytes [56]. As shown in Figure 8e–g, in the KEGG database, the metabolic pathways and purine metabolism pathways are enriched, suggesting that CPAP might activate T cells’ immune functions by regulating purine metabolism [57]. In the Reactome database, four pathways were enriched, including metabolism, the immune system, the innate immune system, and neutrophil degranulation, which indicates that CPAP might alleviate the tumor-induced inflammatory response by promoting neutrophil degranulation and releasing lysozymes and antimicrobial peptides from granules [58]. In the Monarch database, five pathways were enriched, including homeostasis/metabolism phenotype, abnormal homeostasis, immune system phenotype, abnormal immune system physiology, and abnormal response to antigen, which demonstrates that CPAP might enhance antitumor immunity by activating the immune system, improving the metabolic homeostasis of leukocytes, and improving their recognition and response to tumor antigens [59]. Therefore, it can be inferred that CPAP may activate T cells’ antitumor immune functions by regulating purine metabolism and alleviate the tumor-induced inflammatory response by promoting neutrophil degranulation.

### 3.9. Analysis of Highly Expressed Proteins in Solid Tumors from the Model Group

**Figure 9:**
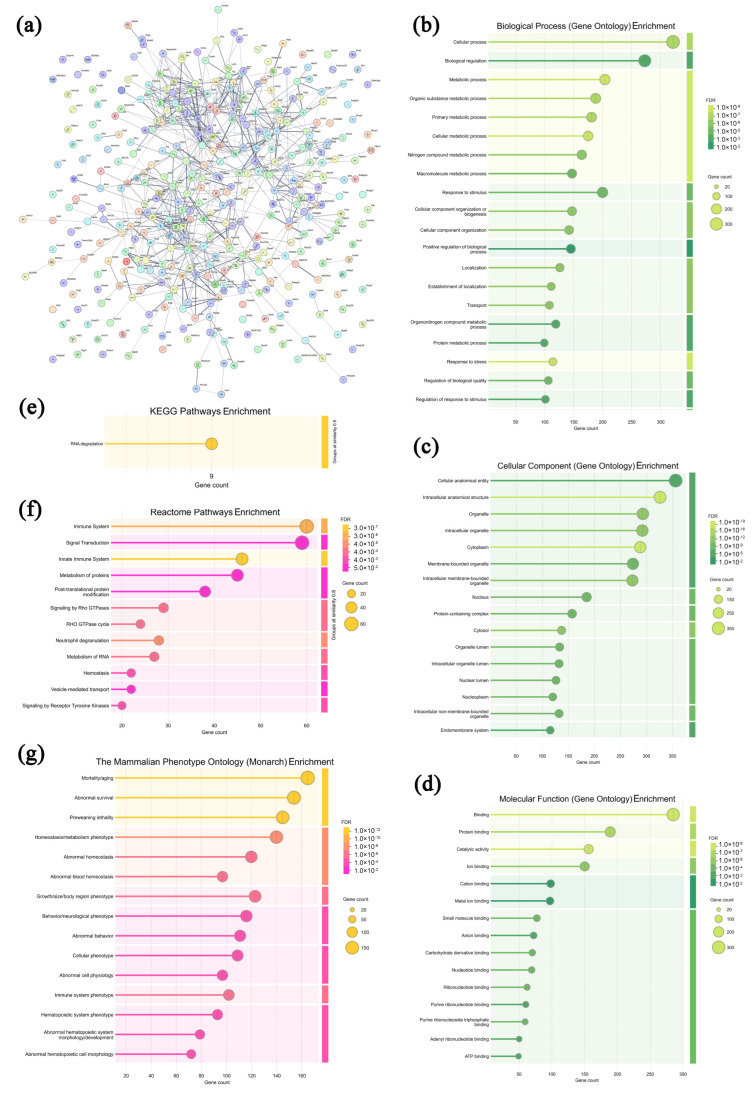
Association network of highly expressed tumor proteins in the model group (**a**) and functional analysis ((**b**–**d**) GO annotations; (**e**–**g**) signaling pathways).

In the solid tumors of the model group, 367 highly expressed proteins were screened and analyzed, and the specific information is shown in Supplementary Table S6. As shown in Figure 9a, most of the highly expressed proteins in the model group exhibited specific interactions, collectively revealing a highly active and finely regulated cellular metabolic and functional network within the TME. In Figure 9b–d, these proteins are primarily enriched in 12 biological processes (the cellular process, biological regulation, the metabolic process, the organic substance metabolic process, the primary metabolic process, etc.), 16 cellular components (the cellular anatomical entity, intracellular anatomical structure, organelle, intracellular organelle, cytoplasm, etc.), and 9 molecular functions (binding, protein binding, catalytic activity, ion binding, cation binding, etc.), suggesting that the tumor cells and infiltrating lymphocytes were in good condition, with intact structures [60]. As displayed in Figure 9e–g, in the KEGG database, the RNA degradation pathway is enriched in the model group, suggesting that the solid tumor cells present characteristics including uncontrolled proliferation, apoptosis resistance, and survival advantage [61]. In the Reactome database, 12 pathways were enriched, including the immune system, signal transduction, the innate immune system, metabolism of proteins, post-translational protein modification, etc., indicating that the roles of the innate immune response in suppressing tumor cell growth were relatively restricted [62]. In the Monarch database, 15 pathways were enriched, including mortality/aging, abnormal survival, preweaning lethality, homeostasis/metabolism phenotype, abnormal homeostasis, etc., indicating that tumor cells underwent biological remodeling to adapt to survival pressures [63]. Therefore, it can be deduced that the solid tumor cells in the model group present structural integrity and uncontrolled proliferation without effective immune response interference.

### 3.10. Analysis of Highly Expressed Proteins in Solid Tumors from the CPAP Group

**Figure 10:**
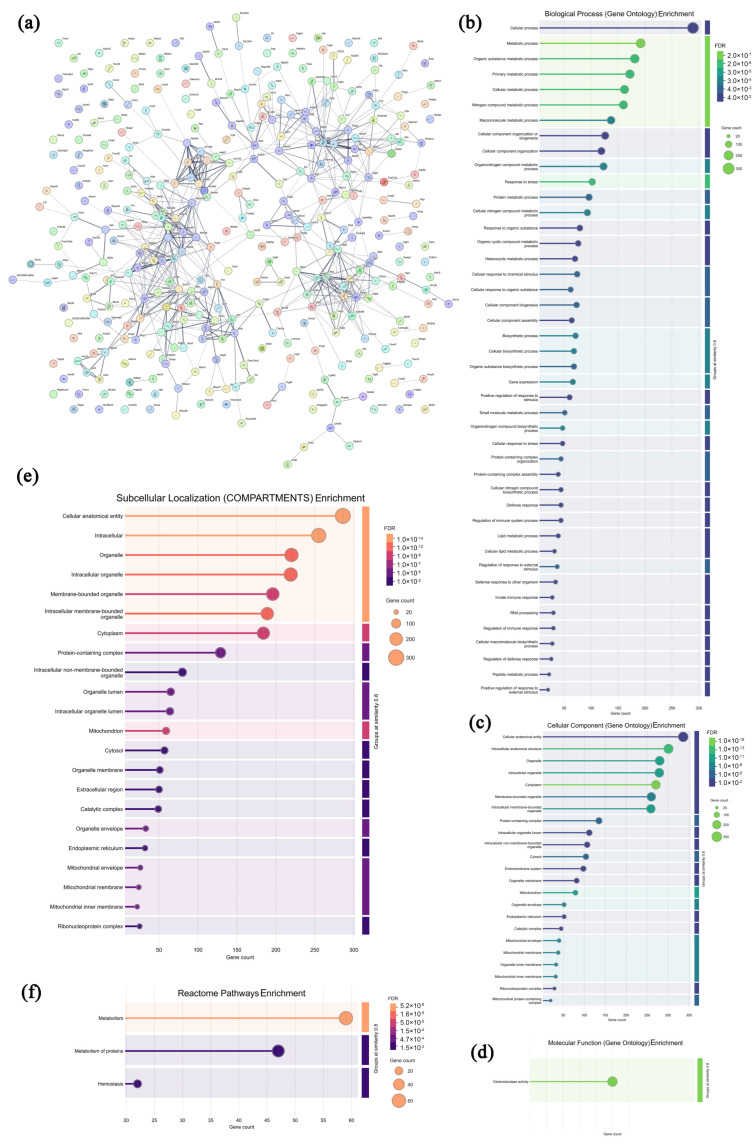
Association network of highly expressed tumor proteins in the CPAP group (**a**) and functional analysis ((**b**–**d**) GO annotations; (**e**,**f**) signaling pathways).

In the solid tumors from the CPAP group, 380 highly expressed proteins were selected and analyzed, and the specific information is presented in Supplementary Table S7. As shown in Figure 10a, most of the highly expressed proteins in the CPAP group exhibited specific interactions, which could form molecular networks with functional synergy. In Figure 10b–d, these proteins are seen to be primarily enriched in 12 biological processes (the cellular process, metabolic process, organic substance metabolic process, primary metabolic process, cellular metabolic process, etc.), 7 cellular components (the cellular anatomical entity, intracellular anatomical structure, organelle, intracellular organelle, cytoplasm, etc.), and 1 molecular function (oxidoreductase activity), indicating that the infiltrating lymphocytes might effectively inhibit the proliferation of tumor cells by regulating the levels of oxidative stress [64]. As shown in Figure 10e,f, in the COMPARTMENTS database, eight pathways were enriched, including the cellular anatomical entity, intracellular organelle, membrane-bounded organelle, intracellular membrane-bounded organelle, cytoplasm, and protein-containing complex, suggesting that CPAP-activated lymphocytes might primarily target the membranes of tumor cells and disrupt the normal physiological processes by affecting organelle function, the cytoplasmic environment, and protein complex interactions [65]. In the Reactome database, three pathways were enriched, including metabolism, metabolism of proteins, and hemostasis, which indicated that CPAP might interfere with the energy supply and biosynthetic processes of tumor cells by modulating various metabolic pathways [66]. Therefore, it can be speculated that CPAP could activate lymphocytes, enable them to recognize and target tumor cells through the membrane, and induce apoptosis in tumor cells by increasing oxidative stress.

## 4. Conclusions

In this study, the regulatory effects of CPAP on gut microbiota metabolism and peripheral blood proteomics in tumor-bearing mice were systematically evaluated. The gut microbiota compositions results showed that CPAP effectively promoted the enrichment of intestinal *Lactobacillus*. In addition, it could be inferred from the metabolomic and proteomic results that CPAP might activate T cells’ antitumor immune functions by regulating purine metabolism, alleviate the tumor-caused inflammatory response by promoting neutrophil degranulation, and induce apoptosis in tumor cells by increasing oxidative stress. These results provide data to support related research on tumor immunity and support further applications in functional food industries.

However, due to the limitations of the technical conditions, intestinal microorganisms can only be identified to the genus level, and the specific *Lactobacillus* species related to antitumor immunity still cannot be determined. Furthermore, the relevant signaling pathways of *Lactobacillus*–immunity–tumor interactions still lack functional validation and require further research, as do the off-target effects of CPAP and the evaluation of consistency from animals to humans. Therefore, future research could focus on the action mechanisms behind CPAP enriching *Lactobacillus* and the signaling pathways activating immune cells to induce apoptosis in tumor cells.

## Data Availability

The original contributions presented in this study are included in the article/Supplementary Materials. Further inquiries can be directed to the corresponding author.

## Supplementary Materials

The following supporting information can be downloaded at: https://www.mdpi.com/article/10.3390/microorganisms13081750/s1, Supplementary Figure S1: Effects of CPPs on the mice body weights (a), immune organ indices (b), tumor weights (c), and tumor inhibitory rates (d). Note: α, *p* < 0.05 compared with blank group; β, *p* < 0.05 compared with model group. Supplementary Table S1: Differentially expressed metabolites of CPAP group compared with the model group. Supplementary Table S2: The information of proteins highly expressed in sera of the model group. Supplementary Table S3: The information of proteins highly expressed in sera of the CPAP group. Supplementary Table S4: The information of proteins highly expressed in leukocytes of the model group. Supplementary Table S5: The information of proteins highly expressed in leukocytes of the CPAP group. Supplementary Table S6: The information of proteins highly expressed in tumors of the model group. Supplementary Table S7: The information of proteins highly expressed in tumors of the CPAP group.
