# Supplementary material for: Regulatory Effects of Codonopsis pilosula Alkali-Extracted Polysaccharide Induced Intestinal Lactobacillus Enrichment on Peripheral Blood Proteomics in Tumor-Bearing Mice
Source: Microorganisms. 2025 Jul 26;13(8):1750. doi: 10.3390/microorganisms13081750 (PMC12388226; doi:10.3390/microorganisms13081750)
Supplement: Supplementary file 1 [file microorganisms-13-01750-s001.zip › microorganisms-3747965-supplementary.docx]

**Supplementary Figures and Tables Captions**

1. Supplementary Figure 1 Effects of CPPs on the mice body weights (a), immune organ indices (b), tumor weights (c), and tumor inhibitory rates (d). Note: α, *p* < 0.05 compared with blank group; β, *p* < 0.05 compared with model group.
2. Supplementary Table 1 Differentially expressed metabolites of CPAP group compared with the model group.
3. Supplementary Table 2 The information of proteins highly expressed in sera of the model group.
4. Supplementary Table 3 The information of proteins highly expressed in sera of the CPAP group.
5. Supplementary Table 4 The information of proteins highly expressed in leukocytes of the model group.
6. Supplementary Table 5 The information of proteins highly expressed in leukocytes of the CPAP group.
7. Supplementary Table 6 The information of proteins highly expressed in tumors of the model group.
8. Supplementary Table 7 The information of proteins highly expressed in tumors of the CPAP group.

**Preliminary experiment results**

**1) Preparation of CPPs**

*C. pilosula* powder was immersed in aqueous solvent with different redox potentials (pH=2, 7 and 12) for polysaccharides extraction (solvent-to-material ratio of 30 mL/g) at 80 °C for 3 h, the extracts were concentrated by a rotating vacuum evaporator and then precipitated with 3 volumes of 95% ethanol for 8 h. Subsequently, the precipitates were dissolved in distilled water, and the insoluble components were removed by centrifugation (6000 g, 10 min). Finally, the CPPs were obtained from the collected supernatant after dialysis (molecular weight cut off of 1000 Da) and lyophilization. Specifically, acidic electrolyzed water (pH 2, +1100 mV), deionized water (pH 7), and alkaline electrolyzed water (pH 12, −1100 mV) were applied respectively for the CPP2, CPP7, and CPP12 preparation. **CPP12 was also named *C. pilosula* alkali-extracted polysaccharides (CPAP) in this paper (microorganisms-3747965).**

**2) Animal experimental design of CPPs**

The mice were randomly divided into 9 groups (n = 10/group): blank, model, cyclophosphamide (CTX), and CPP2, CPP7, CPP12 groups (low-dose of 50 mg/kg, high-dose of 100 mg/kg). After 1 week acclimatization period, mice received daily oral gavage for 1 week: blank, model, and CTX groups were administered 0.2 mL saline, while the CPPs groups received equivalent volumes of polysaccharide solution. Subsequently, S180 tumor cells of 0.2 mL (5 × 10⁶ cells/mouse) were subcutaneously inoculated into the right axilla of all groups except the blank group. The CTX group began intraperitoneally injection with CTX (30 mg/kg/d), while other groups continued gavage treatments for another 14 days.

**3) Physiological indicators collection**

At the end of the experimental period, mice were euthanized via cervical dislocation. Spleens, thymuses, and solid tumors were excised and weighed. Organ indices (mg/g) were calculated as immune organ weight (mg) / body weight (g). Tumor inhibition rate (%) was determined as [1 − (tumor weight of treatment group / tumor weight of model group)] × 100.

**4) Results**

The immunomodulatory effects of CPPs were evaluated in tumor-bearing mice, and the physiological indices determination results were shown in Supplementary Figure 1. As presented in Supplementary Figure 1 (a), compared with the model group, CTX treatment significantly reduced the body weights (*p* < 0.05) of tumor-bearing mice, which could be attributed to the digestive system damage induced impaired nutrient absorption, metabolic disturbances induced elevated energy expenditure, and the systemic toxicity on both tumors and immune system. In contrast, CPPs groups maintained body weights well compared with the blank group, suggesting improved systemic health and potential antitumor efficacy. As demonstrated in Supplementary Figure 1 (b), the spleen indices in the model group were markedly ascended (*p* < 0.05) compared with those of the blank group, while the thymus indices were significantly decreased (*p* < 0.05), indicating the tumor-induced splenomegaly, thymus atrophy and compromised immune functions. CPPs groups significantly reduced spleen indices (*p* < 0.05) and improved the thymus indices (*p* < 0.05) compared with the model group, demonstrating the protective effects on these immune organs. Tumor weights and inhibition rates determination results were shown in Supplementary Figure 1 (c) and (d). Compared with the model group, CTX and CPP treatments all significantly reduced the tumor weights (*p* < 0.05). The high dose of CPPs groups exhibited the inhibition rates of 33.17% (CPP2), 51.63% (CPP7), and 59.52% (CPP12), respectively, demonstrating that the CPP12 presented the highest antitumor efficacy, even surpassing the inhibitory rates of CTX (52.88%). CTX indiscriminately suppressed immune cells and tumor cells, whereas CPP12 eliminated tumor cells *in vivo* via the immune system activation.


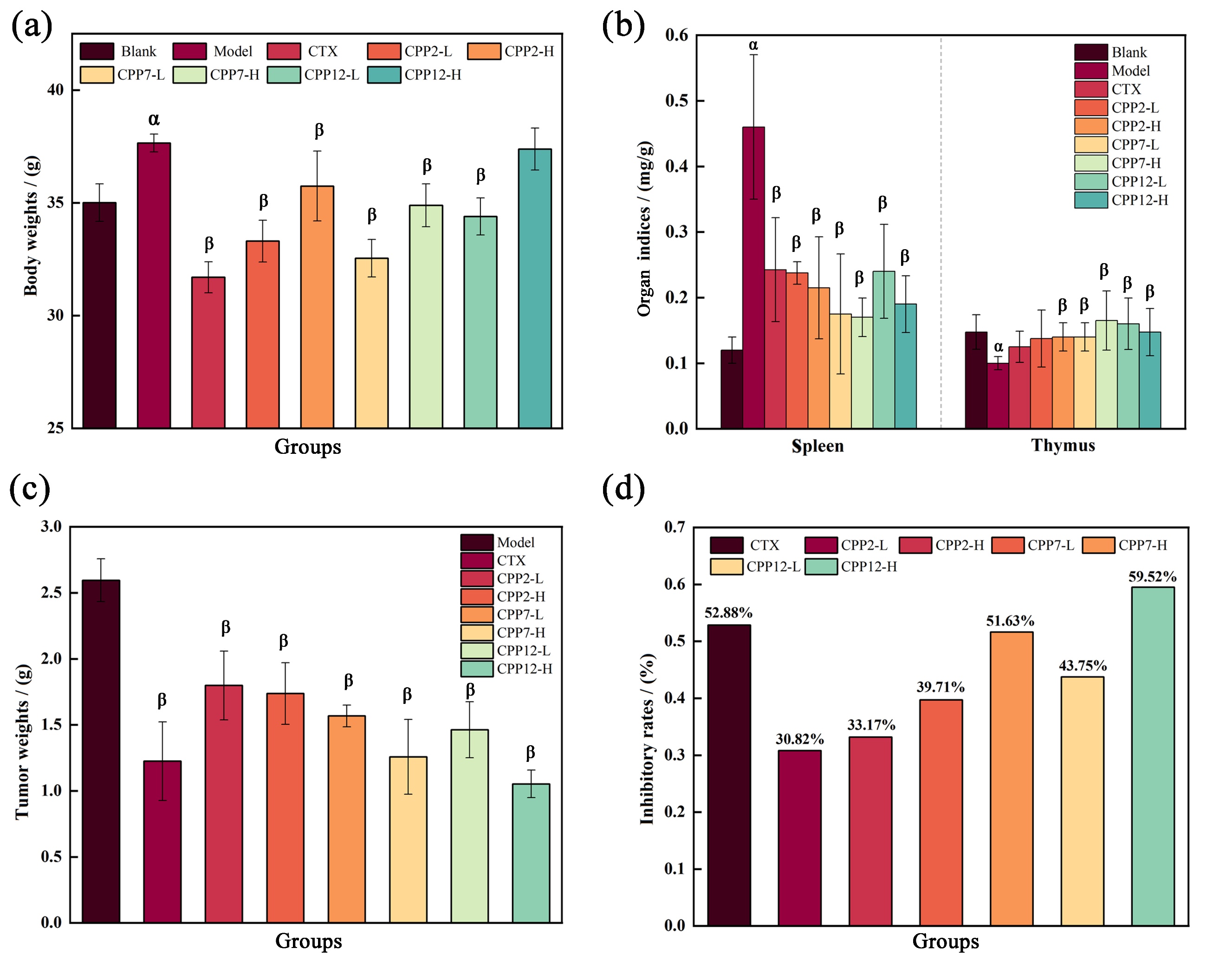


**Supplementary Figure S1** **Effects of CPPs on the mice body weights (a), immune organ indices (b), tumor weights (c), and tumor inhibitory rates (d).**

**Note:** **α, *p* < 0.05 compared with blank group; β, *p* < 0.05 compared with model group.**

**It needs to be clarified that Supplementary Figure 1 shows the shared basic data of animal experiments used by this study and another manuscript under review (****Fig. 6, Manuscript Number: INDCRO-D-25-02306R3; Title: Structural characteristics and intestinal immunity of *Codonopsis pilosula* polysaccharides prepared by electrolytic water; Status: With editor; Status Date: Jul 16, 2025).** **To ensure that readers can accurately understand the data when making cross-study comparisons and to avoid any ambiguity, this figure is consistent with the corresponding Fig. 6 in the above-mentioned manuscript under review.**

**Supplementary Table S1 Differentially expressed metabolites of CPAP group compared with the model group.**

| **ID** | **Description** | **Retention time(min)** | | **m/z** | **log2FC** | **P-value** | **Up or Down** |
| --- | --- | --- | --- | --- | --- | --- | --- |
| C589 | LPC14:0 | | 8.60 | 466.29 | -3.99 | 0.02 | down |
| C48 | 1-(4-methyl-2-pyridyl)pyrrolidine-2,5-dione | | 1.00 | 191.08 | -8.55 | 0.03 | down |
| C302 | ACar18:3 | | 7.06 | 423.33 | -3.90 | 0.00 | down |
| C770 | PC(15:0/16:0) | | 14.09 | 720.55 | -3.68 | 0.03 | down |
| C1187 | dimethyl2-(3-nitro-2-pyridyl)malonate | | 4.86 | 255.06 | -5.04 | 0.04 | down |
| C1013 | Uridine | | 0.58 | 243.06 | -2.62 | 0.01 | down |
| C466 | Ecgoninemethylester | | 3.83 | 200.13 | -1.58 | 0.01 | down |
| C364 | Carvone | | 13.02 | 151.11 | -1.57 | 0.02 | down |
| C444 | Dl-Citrulline | | 13.02 | 176.10 | -1.63 | 0.03 | down |
| C387 | Citrulline | | 10.78 | 176.10 | -1.89 | 0.02 | down |
| C699 | N2-ethyl-N4-isopropyl-6-(methylthio)-1,3,5-triazine-2,4-diamine | | 11.23 | 228.13 | -2.09 | 0.00 | down |
| C722 | N-Heptanoylhomoserinelactone | | 2.43 | 214.14 | -1.80 | 0.04 | down |
| C862 | PC(O-16:0/18:2(9Z,12Z)) | | 0.52 | 744.59 | -2.49 | 0.02 | down |
| C38 | 1-(2,6-difluorobenzyl)piperidinehydrochloride | | 0.53 | 212.12 | -5.45 | 0.02 | down |
| C1000 | TQH | | 7.63 | 385.18 | -1.97 | 0.02 | down |
| C256 | 6-(4-phenylpiperazino)hexanoicacidhydrochloride | | 5.51 | 277.19 | -3.06 | 0.01 | down |
| C928 | RMK | | 7.87 | 434.25 | 7.97 | 0.00 | up |
| C511 | Glycerophospho-N-palmitoylethanolamine | | 6.66 | 452.28 | 4.42 | 0.05 | up |
| C1027 | (2,3,9,17,22R)-2,3,14,20,22-Pentahydroxyergost-7-en-6-one | | 6.28 | 479.34 | 5.11 | 0.02 | up |
| C320 | Adenine | | 0.60 | 134.05 | 1.56 | 0.03 | up |
| C1026 | (1,2,3,5,9,18)-1,2,3,19-Tetrahydroxyurs-12-en-28-oicacid | | 7.46 | 505.35 | 4.38 | 0.01 | up |
| C418 | Dehydrocholicacid | | 4.75 | 403.25 | 2.70 | 0.03 | up |
| C542 | Hyodeoxycholicacid | | 7.95 | 393.30 | 2.78 | 0.01 | up |
| C188 | 3-Hydroxy-cis-5-tetradecenoylcarnitine | | 8.61 | 386.29 | 1.63 | 0.02 | up |
| C318 | Actrarit | | 1.03 | 194.08 | 3.29 | 0.01 | up |
| C223 | 4-Anilino-4-oxobutanoicacid | | 1.45 | 194.08 | 3.22 | 0.01 | up |
| C1036 | 18--Glycyrrhetinicacid | | 9.39 | 471.35 | 2.08 | 0.01 | up |
| C614 | LPE22:5 | | 7.06 | 528.31 | 2.29 | 0.04 | up |
| C714 | NAT13-331713_POS | | 14.34 | 447.24 | 3.36 | 0.02 | up |
| C194 | 3-ketoFusidicacid | | 7.38 | 529.32 | 1.73 | 0.01 | up |
| C825 | PC(19:1/20:5) | | 7.98 | 820.59 | 4.01 | 0.04 | up |

**Supplementary Table S2 The information of proteins highly expressed in sera of the model group.**

| **Protein** | **Description** | **Gene** | **Protein** | **Description** | **Gene** |
| --- | --- | --- | --- | --- | --- |
| Q61702 | Inter-alpha-trypsin inhibitor heavy chain H1 | Itih1 | P12246 | Serum amyloid P-component | Apcs |
| P17742 | Peptidyl-prolyl cis-trans isomerase A | Ppia | Q61083 | Mitogen-activated protein kinase kinase kinase 2 | Map3k2 |
| P10126 | Elongation factor 1-alpha 1 | Eef1a1 | O09061 | Proteasome subunit beta type-1 | Psmb1 |
| Q63805 | Alpha-1-acid glycoprotein 3 | Orm3 | P05064 | Fructose-bisphosphate aldolase A | Aldoa |
| Q64442 | Sorbitol dehydrogenase | Sord | P26041 | Moesin | Msn |
| O35664 | Interferon alpha/beta receptor 2 | Ifnar2 | B2RPV6 | Multimerin-1 | Mmrn1 |
| Q61147 | Ceruloplasmin | Cp | O88379 | Bromodomain adjacent to zinc finger domain protein 1A | Baz1a |
| O08677 | Kininogen-1 | Kng1 | Q9R1P4 | Proteasome subunit alpha type-1 | Psma1 |
| Q9JHH6 | Carboxypeptidase B2 | Cpb2 | P18531 | Ig heavy chain V region 3-6 | Ighv3-6 |
| Q8CIZ8 | von Willebrand factor | Vwf | Q99K28 | ADP-ribosylation factor GTPase-activating protein 2 | Arfgap2 |
| Q61805 | Lipopolysaccharide-binding protein | Lbp | P17182 | Alpha-enolase | Eno1 |
| P20029 | Endoplasmic reticulum chaperone BiP | Hspa5 | P07758 | Alpha-1-antitrypsin 1-1 | Serpina1a |
| O09131 | Glutathione S-transferase omega-1 | Gsto1 | P05367 | Serum amyloid A-2 protein | Saa2 |
| Q8K0E8 | Fibrinogen beta chain | Fgb | P63017 | Heat shock cognate 71 kDa protein | Hspa8 |
| O88844 | Isocitrate dehydrogenase [NADP] cytoplasmic | Idh1 | Q9R1P0 | Proteasome subunit alpha type-4 | Psma4 |
| P70375 | Coagulation factor VII | F7 | Q60692 | Proteasome subunit beta type-6 | Psmb6 |
| P61939 | Thyroxine-binding globulin | Serpina7 | Q9DBJ1 | Phosphoglycerate mutase 1 | Pgam1 |
| P05366 | Serum amyloid A-1 protein | Saa1 | Q9QZ25 | Vascular non-inflammatory molecule 3 | Vnn3 |
| O70410 | Vomeronasal type-2 receptor 1 | Vmn2r1 | P40142 | Transketolase | Tkt |
| P60710 | Actin, cytoplasmic 1 | Actb | P68033 | Actin, alpha cardiac muscle 1 | Actc1 |
| Q71KU9 | Fibrinogen-like protein 1 | Fgl1 | Q9QUM9 | Proteasome subunit alpha type-6 | Psma6 |
| P11672 | Neutrophil gelatinase-associated lipocalin | Lcn2 | Q9JM99 | Proteoglycan 4 | Prg4 |
| P28076 | Proteasome subunit beta type-9 | Psmb9 | Q93092 | Transaldolase | Taldo1 |
| Q60590 | Alpha-1-acid glycoprotein 1 | Orm1 | P10923 | Osteopontin | Spp1 |
| Q61704 | Inter-alpha-trypsin inhibitor heavy chain H3 | Itih3 | Q9Z2U0 | Proteasome subunit alpha type-7 | Psma7 |
| P10605 | Cathepsin B | Ctsb | P62259 | 14-3-3 protein epsilon | Ywhae |
| Q19LI2 | Alpha-1B-glycoprotein | A1bg | O55234 | Proteasome subunit beta type-5 | Psmb5 |
| P14152 | Malate dehydrogenase, cytoplasmic | Mdh1 | P01657 | Ig kappa chain V-III region PC 2413 | |
| A6X935 | Inter alpha-trypsin inhibitor, heavy chain 4 | Itih4 | Q6GQT1 | Alpha-2-macroglobulin-P | A2m |
| P21550 | Beta-enolase | Eno3 | O70435 | Proteasome subunit alpha type-3 | Psma3 |
| P08905 | Lysozyme C-2 | Lyz2 | P24527 | Leukotriene A-4 hydrolase | Lta4h |
| Q8BTM8 | Filamin-A | Flna | P17751 | Triosephosphate isomerase | Tpi1 |
| Q9R1P3 | Proteasome subunit beta type-2 | Psmb2 | P35700 | Peroxiredoxin-1 | Prdx1 |
| P00920 | Carbonic anhydrase 2 | Ca2 | P10810 | Monocyte differentiation antigen CD14 | Cd14 |
| Q64727 | Vinculin | Vcl | Q99K85 | Phosphoserine aminotransferase | Psat1 |
| P52480 | Pyruvate kinase PKM | Pkm | P58252 | Elongation factor 2 | Eef2 |
| Q9DCD0 | 6-phosphogluconate dehydrogenase, decarboxylating | Pgd | P09411 | Phosphoglycerate kinase 1 | Pgk1 |
| Q9Z2U1 | Proteasome subunit alpha type-5 | Psma5 | P16045 | Galectin-1 | Lgals1 |
| P07361 | Alpha-1-acid glycoprotein 2 | Orm2 | P68510 | 14-3-3 protein eta | Ywhah |
| Q07456 | Protein AMBP | Ambp | P61982 | 14-3-3 protein gamma | Ywhag |
| Q91WP6 | Serine protease inhibitor A3N | Serpina3n | P40124 | Adenylyl cyclase-associated protein 1 | Cap1 |
| P06151 | L-lactate dehydrogenase A chain | Ldha | P28063 | Proteasome subunit beta type-8 | Psmb8 |
| P11499 | Heat shock protein HSP 90-beta | Hsp90ab1 | Q8R016 | Bleomycin hydrolase | Blmh |
| P06745 | Glucose-6-phosphate isomerase | Gpi | P04918 | Serum amyloid A-3 protein | Saa3 |
| Q61316 | Heat shock 70 kDa protein 4 | Hspa4 | A0JNU3 | 60 kDa lysophospholipase | Aspg |
| P99026 | Proteasome subunit beta type-4 | Psmb4 | P48678 | Prelamin-A/C | Lmna |
| P63101 | 14-3-3 protein zeta/delta | Ywhaz | P27546 | Microtubule-associated protein 4 | Map4 |
| Q64323 | Phosphatidylinositol N-acetylglucosaminyltransferase subunit A | Piga | Q9CXX0 | Centriolar and ciliogenesis-associated protein HYLS1 | Hyls1 |
| P26040 | Ezrin | Ezr | Q3UHU5 | Microtubule cross-linking factor 1 | Mtcl1 |
| Q01768 | Nucleoside diphosphate kinase B | Nme2 | A1Z198 | NACHT, LRR and PYD domains-containing protein 1b allele 2 | Nlrp1b |

**Supplementary Table S3 The information of proteins highly expressed in sera of the CPAP group.**

| **Protein** | **Description** | **Gene** | **Protein** | **Description** | **Gene** |
| --- | --- | --- | --- | --- | --- |
| Q8BK48 | Pyrethroid hydrolase Ces2e | Ces2e | Q80YC5 | Coagulation factor XII | F12 |
| P01592 | Immunoglobulin J chain | Jchain | P05208 | Chymotrypsin-like elastase family member 2A | Cela2a |
| Q9CQF9 | Prenylcysteine oxidase 1 | Pcyox1 | P04104 | Keratin, type II cytoskeletal 1 | Krt1 |
| Q9QWK4 | CD5 antigen-like | Cd5l | Q9ET66 | Peptidase inhibitor 16 | Pi16 |
| Q00897 | Alpha-1-antitrypsin 1-4 | Serpina1d | Q8VCG4 | Complement component C8 gamma chain | C8g |
| P13020 | Gelsolin | Gsn | P70274 | Selenoprotein P | Selenop |
| P31532 | Serum amyloid A-4 protein | Saa4 | P51885 | Lumican | Lum |
| P28798 | Progranulin | Grn | P26262 | Plasma kallikrein | Klkb1 |
| Q07079 | Insulin-like growth factor-binding protein 5 | Igfbp5 | P02088 | Hemoglobin subunit beta-1 | Hbb-b1 |
| Q01339 | Beta-2-glycoprotein 1 | Apoh | P01787 | Ig heavy chain V regions TEPC 15/S107/HPCM1/HPCM2/HPCM3 |  |
| Q8BND5 | Sulfhydryl oxidase 1 | Qsox1 | P41317 | Mannose-binding protein C | Mbl2 |
| Q9CQW3 | Vitamin K-dependent protein Z | Proz | P34928 | Apolipoprotein C-I | Apoc1 |
| O70362 | Phosphatidylinositol-glycan-specific phospholipase D | Gpld1 | P16882 | Growth hormone receptor | Ghr |
| Q8CG14 | Complement C1s-1 subcomponent | C1s1 | P43025 | Tetranectin | Clec3b |
| Q8K182 | Complement component C8 alpha chain | C8a | Q9CR35 | Chymotrypsinogen B | Ctrb1 |
| P82198 | Transforming growth factor-beta-induced protein ig-h3 | Tgfbi | P01867 | Immunoglobulin heavy constant gamma 2B | Ighg2b |
| P01801 | Ig heavy chain V-III region J606 |  | P07309 | Transthyretin | Ttr |
| P18528 | Ig heavy chain V region 6.96 |  | Q06770 | Corticosteroid-binding globulin | Serpina6 |
| P22599 | Alpha-1-antitrypsin 1-2 | Serpina1b | Q5SPW0 | Vacuolar protein sorting-associated protein 54 | Vps54 |
| P06683 | Complement component C9 | C9 | P01864 | Ig gamma-2A chain C region secreted form |  |
| P18525 | Ig heavy chain V region 5-84 |  | P01819 | Ig heavy chain V region MOPC 141 |  |
| P04247 | Myoglobin | Mb | O89020 | Afamin | Afm |
| P23953 | Carboxylesterase 1C | Ces1c | P01865 | Ig gamma-2A chain C region, membrane-bound form | Igh-1a |
| Q9R182 | Angiopoietin-related protein 3 | Angptl3 | P42703 | Leukemia inhibitory factor receptor | Lifr |
| Q9D3R6 | Katanin p60 ATPase-containing subunit A-like 2 | Katnal2 | P01869 | Ig gamma-1 chain C region, membrane-bound form | Ighg1 |
| Q8BH35 | Complement component C8 beta chain | C8b | P01878 | Ig alpha chain C region |  |
| P01750 | Ig heavy chain V region 102 |  | Q9WVJ3 | Carboxypeptidase Q | Cpq |
| Q8K0D2 | Hyaluronan-binding protein 2 | Habp2 | P04939 | Major urinary protein 3 | Mup3 |
| P00687 | Alpha-amylase 1 | Amy1 | P01897 | H-2 class I histocompatibility antigen, L-D alpha chain | H2-L |
| Q61730 | Interleukin-1 receptor accessory protein | Il1rap | P01746 | Ig heavy chain V region 93G7 |  |
| P21614 | Vitamin D-binding protein | Gc | P02535 | Keratin, type I cytoskeletal 10 | Krt10 |
| P13595 | Neural cell adhesion molecule 1 | Ncam1 | P11087 | Collagen alpha-1(I) chain | Col1a1 |
| P18524 | Ig heavy chain V region RF |  | Q00724 | Retinol-binding protein 4 | Rbp4 |
| P21460 | Cystatin-C | Cst3 | P04945 | Ig kappa chain V-VI region NQ2-6.1 |  |
| P01725 | Ig lambda-1 chain V region S178 |  | O09164 | Extracellular superoxide dismutase [Cu-Zn] | Sod3 |
| Q08879 | Fibulin-1 | Fbln1 | P01749 | Ig heavy chain V region 3 | Ighv1-61 |
| Q9Z0M6 | Adhesion G protein-coupled receptor E5 | Adgre5 | P01629 | Ig kappa chain V-II region 2S1.3 |  |
| Q9ES30 | Complement C1q tumor necrosis factor-related protein 3 | C1qtnf3 | P18337 | L-selectin | Sell |
| Q8BVA4 | Leiomodin-1 | Lmod1 |  |  |  |

**Supplementary Table S4 The information of proteins highly expressed in leukocytes of the model group.**

| **Protein** | **Description** | **Gene** | **Protein** | **Description** | **Gene** | **Protein** | **Description** | **Gene** |
| --- | --- | --- | --- | --- | --- | --- | --- | --- |
| P36423 | Thromboxane-A synthase | Tbxas1 | Q9Z2P8 | Vesicle-associated membrane protein 5 | Vamp5 | Q5XJY4 | Presenilin-associated rhomboid-like protein, mitochondrial | Parl |
| Q8K1K6 | Serpin B10 | Serpinb10 | Q60738 | Proton-coupled zinc antiporter SLC30A1 | Slc30a1 | Q61151 | Serine/threonine-protein phosphatase 2A 56 kDa regulatory subunit epsilon isoform | Ppp2r5e |
| O55143 | Sarcoplasmic/endoplasmic reticulum calcium ATPase 2 | Atp2a2 | Q91VS7 | Microsomal glutathione S-transferase 1 | Mgst1 | A1L314 | Macrophage-expressed gene 1 protein | Mpeg1 |
| Q62095 | ATP-dependent RNA helicase DDX3Y | Ddx3y | Q9DCF9 | Translocon-associated protein subunit gamma | Ssr3 | P11531 | Dystrophin | Dmd |
| Q9DBG6 | Dolichyl-diphosphooligosaccharide--protein glycosyltransferase subunit 2 | Rpn2 | Q8C7X2 | ER membrane protein complex subunit 1 | Emc1 | Q762D5 | UDP-N-acetylglucosamine/UDP-glucose/GDP-mannose transporter | Slc35d2 |
| Q8R5C5 | Beta-centractin | Actr1b | Q5SWT3 | Solute carrier family 25 member 35 | Slc25a35 | Q8C0Q4 | Serine/threonine-protein kinase Nek11 | Nek11 |
| P70704 | Phospholipid-transporting ATPase IA | Atp8a1 | Q9CZG3 | COMM domain-containing protein 8 | Commd8 | Q8C0L8 | Conserved oligomeric Golgi complex subunit 5 | Cog5 |
| P68433 | Histone H3.1 | H3c1 | Q9JKF7 | Large ribosomal subunit protein mL39 | Mrpl39 | Q5SS00 | DBF4-type zinc finger-containing protein 2 homolog | Zdbf2 |
| P29387 | Guanine nucleotide-binding protein subunit beta-4 | Gnb4 | P43406 | Integrin alpha-V | Itgav | Q7TSY8 | Shugoshin 2 | Sgo2 |
| A1L3T7 | RIPOR family member 3 | Ripor3 | Q8K1C0 | Protein angel homolog 2 | Angel2 | Q9D168 | Integrator complex subunit 12 | Ints12 |
| Q61143 | Short transient receptor potential channel 6 | Trpc6 | Q68FF6 | ARF GTPase-activating protein GIT1 | Git1 | Q52KR2 | Leucine-rich repeats and immunoglobulin-like domains protein 2 | Lrig2 |
| Q8BY89 | Choline transporter-like protein 2 | Slc44a2 | Q8K2Q7 | BRO1 domain-containing protein BROX | Brox | P51830 | Adenylate cyclase type 9 | Adcy9 |
| Q80WJ7 | Protein LYRIC | Mtdh | Q60857 | Sodium-dependent serotonin transporter | Slc6a4 | Q61941 | NAD(P) transhydrogenase, mitochondrial | Nnt |
| Q80XR2 | Calcium-transporting ATPase type 2C member 1 | Atp2c1 | Q9D1E8 | 1-acyl-sn-glycerol-3-phosphate acyltransferase epsilon | Agpat5 | P60761 | Neurogranin | Nrgn |
| Q6URW6 | Myosin-14 | Myh14 | P15702 | Leukosialin | Spn | Q5SSH7 | Zinc finger ZZ-type and EF-hand domain-containing protein 1 | Zzef1 |
| Q91YE6 | Importin-9 | Ipo9 | P48545 | G protein-activated inward rectifier potassium channel 4 | Kcnj5 | Q8K012 | Formin-binding protein 1-like | Fnbp1l |
| Q80ZJ1 | Ras-related protein Rap-2a | Rap2a | P52875 | Transmembrane protein 165 | Tmem165 | Q99P31 | Hsp70-binding protein 1 | Hspbp1 |
| Q9CQD1 | Ras-related protein Rab-5A | Rab5a | Q99PV0 | Pre-mRNA-processing-splicing factor 8 | Prpf8 | Q921M3 | Splicing factor 3B subunit 3 | Sf3b3 |
| Q9QYA2 | Mitochondrial import receptor subunit TOM40 homolog | Tomm40 | Q9D5T0 | Outer mitochondrial transmembrane helix translocase | Atad1 | Q2NL51 | Glycogen synthase kinase-3 alpha | Gsk3a |
| O70503 | Very-long-chain 3-oxoacyl-CoA reductase | Hsd17b12 | Q60759 | Glutaryl-CoA dehydrogenase, mitochondrial | Gcdh | Q8R1I1 | Cytochrome b-c1 complex subunit 9 | Uqcr10 |
| Q9JIY5 | Serine protease HTRA2, mitochondrial | Htra2 | Q9DB43 | Zinc finger protein-like 1 | Zfpl1 | Q9D517 | 1-acyl-sn-glycerol-3-phosphate acyltransferase gamma | Agpat3 |
| Q9QXK3 | Coatomer subunit gamma-2 | Copg2 | Q3U3Q1 | Serine/threonine-protein kinase ULK3 | Ulk3 | Q03249 | Galactose-1-phosphate uridylyltransferase | Galt |
| P11370 | Retrovirus-related Env polyprotein from Fv-4 locus | Fv4 | Q3TFD2 | Lysophosphatidylcholine acyltransferase 1 | Lpcat1 | P21958 | Antigen peptide transporter 1 | Tap1 |
| Q6X893 | Choline transporter-like protein 1 | Slc44a1 | Q9JJF9 | Signal peptide peptidase-like 2A | Sppl2a | Q99N96 | Large ribosomal subunit protein uL1m | Mrpl1 |
| Q6PDI5 | Proteasome adapter and scaffold protein ECM29 | Ecpas | Q64337 | Sequestosome-1 | Sqstm1 | Q9QXN3 | Activating signal cointegrator 1 | Trip4 |
| Q9D1J1 | Adaptin ear-binding coat-associated protein 2 | Necap2 | Q8BZW8 | NHL repeat-containing protein 2 | Nhlrc2 | Q9QXB9 | Developmentally-regulated GTP-binding protein 2 | Drg2 |
| O54774 | AP-3 complex subunit delta-1 | Ap3d1 | Q3UJU9 | Regulator of microtubule dynamics protein 3 | Rmdn3 | Q9QZM0 | Ubiquilin-2 | Ubqln2 |
| Q9DC51 | Guanine nucleotide-binding protein G(i) subunit alpha-3 | Gnai3 | O35435 | Dihydroorotate dehydrogenase (quinone), mitochondrial | Dhodh | Q60865 | Caprin-1 | Caprin1 |
| Q2TPA8 | Hydroxysteroid dehydrogenase-like protein 2 | Hsdl2 | Q9R0E1 | Multifunctional procollagen lysine hydroxylase and glycosyltransferase LH3 | Plod3 | Q923T9 | Calcium/calmodulin-dependent protein kinase type II subunit gamma | Camk2g |
| Q9R1J0 | Sterol-4-alpha-carboxylate 3-dehydrogenase, decarboxylating | Nsdhl | P57787 | Monocarboxylate transporter 4 | Slc16a3 | P04441 | H-2 class II histocompatibility antigen gamma chain | Cd74 |
| Q8BX70 | Intermembrane lipid transfer protein VPS13C | Vps13c | Q99LG2 | Transportin-2 | Tnpo2 | Q9D8B4 | NADH dehydrogenase [ubiquinone] 1 alpha subcomplex subunit 11 | Ndufa11 |
| Q9ET54 | Palladin | Palld | Q8C863 | E3 ubiquitin-protein ligase Itchy | Itch | P70224 | GTPase IMAP family member 1 | Gimap1 |
| P61620 | Protein transport protein Sec61 subunit alpha isoform 1 | Sec61a1 | Q9CQU3 | Protein RER1 | Rer1 | Q9EQ20 | Methylmalonate-semialdehyde dehydrogenase [acylating], mitochondrial | Aldh6a1 |
| Q91VN4 | MICOS complex subunit Mic25 | Chchd6 | O35972 | Large ribosomal subunit protein uL23m | Mrpl23 | Q9DC70 | NADH dehydrogenase [ubiquinone] iron-sulfur protein 7, mitochondrial | Ndufs7 |
| Q3TDQ1 | Dolichyl-diphosphooligosaccharide--protein glycosyltransferase subunit STT3B | Stt3b | P01662 | Ig kappa chain V-III region ABPC 22/PC 9245 | | Q8BHA3 | D-aminoacyl-tRNA deacylase 2 | Dtd2 |
| Q9CYN2 | Signal peptidase complex subunit 2 | Spcs2 | P70280 | Vesicle-associated membrane protein 7 | Vamp7 | Q9CPV9 | P2Y purinoceptor 12 | P2ry12 |
| P10810 | Monocyte differentiation antigen CD14 | Cd14 | Q80U63 | Mitofusin-2 | Mfn2 | P69566 | Ran-binding protein 9 | Ranbp9 |
| Q61584 | RNA-binding protein FXR1 | Fxr1 | P03888 | NADH-ubiquinone oxidoreductase chain 1 | Mtnd1 | Q8BK08 | Transmembrane protein 11, mitochondrial | Tmem11 |
| P01902 | H-2 class I histocompatibility antigen, K-D alpha chain | H2-K1 | Q8BYA0 | Tubulin-specific chaperone D | Tbcd | O35382 | Exocyst complex component 4 | Exoc4 |
| Q8VBT0 | Thioredoxin-related transmembrane protein 1 | Tmx1 | Q80WQ2 | Protein VAC14 homolog | Vac14 | Q08351 | Thrombopoietin receptor | Mpl |
| Q9EP69 | Phosphatidylinositol-3-phosphatase SAC1 | Sacm1l | Q8CG50 | Ras-related protein Rab-43 | Rab43 | Q9JIM1 | Equilibrative nucleoside transporter 1 | Slc29a1 |
| P48024 | Eukaryotic translation initiation factor 1 | Eif1 | Q9Z0S4 | Claudin-13 | Cldn13 | Q9EP72 | ER membrane protein complex subunit 7 | Emc7 |
| Q3UNZ8 | Quinone oxidoreductase-like protein 2 | Cryzl2 | Q8CDM8 | FHF complex subunit HOOK interacting protein 2A | Fhip2a | Q9Z130 | Heterogeneous nuclear ribonucleoprotein D-like | Hnrnpdl |
| Q9CZ42 | ATP-dependent (S)-NAD(P)H-hydrate dehydratase | Naxd | Q78XF5 | Oligosaccharyltransferase complex subunit OSTC | Ostc | P51807 | Dynein light chain Tctex-type 1 | Dynlt1 |
| Q8BH02 | Torsin-4A | Tor4a | Q9Z1Q4 | Lymphocyte antigen 6 complex locus protein G6c | Ly6g6c | Q8BRU6 | Synaptic vesicular amine transporter | Slc18a2 |
| Q8BHZ0 | CYFIP-related Rac1 interactor A | Cyria | Q8CIG8 | Protein arginine N-methyltransferase 5 | Prmt5 | Q9QUJ7 | Long-chain-fatty-acid--CoA ligase 4 | Acsl4 |
| A0A140LIF8 | Immunity-related GTPase family M protein 2 | Irgm2 | P30355 | Arachidonate 5-lipoxygenase-activating protein | Alox5ap | Q8R2R9 | AP-3 complex subunit mu-2 | Ap3m2 |
| Q6GQT9 | BOS complex subunit NOMO1 | Nomo1 | Q8BKE6 | Cytochrome P450 20A1 | Cyp20a1 | Q99KK2 | N-acylneuraminate cytidylyltransferase | Cmas |
| Q80UM7 | Mannosyl-oligosaccharide glucosidase | Mogs | Q3UHQ6 | Protein dopey-2 | Dop1b | Q9D8V0 | Minor histocompatibility antigen H13 | Hm13 |
| Q9ET30 | Transmembrane 9 superfamily member 3 | Tm9sf3 | Q6KAR6 | Exocyst complex component 3 | Exoc3 | P83093 | Stromal interaction molecule 2 | Stim2 |
| Q9CRD2 | ER membrane protein complex subunit 2 | Emc2 | Q9CR61 | NADH dehydrogenase [ubiquinone] 1 beta subcomplex subunit 7 | Ndufb7 | Q3UIR3 | E3 ubiquitin-protein ligase DTX3L | Dtx3l |
| Q9ESW4 | Acylglycerol kinase, mitochondrial | Agk | Q8BJU2 | Tetraspanin-9 | Tspan9 | P40224 | Stromal cell-derived factor 1 | Cxcl12 |
| Q9JJK2 | LanC-like protein 2 | Lancl2 | Q9JMD3 | START domain-containing protein 10 | Stard10 | Q8K2C9 | Very-long-chain (3R)-3-hydroxyacyl-CoA dehydratase 3 | Hacd3 |
| Q04750 | DNA topoisomerase 1 | Top1 | Q99K23 | Ufm1-specific protease 2 | Ufsp2 | Q6PAV2 | Probable E3 ubiquitin-protein ligase HERC4 | Herc4 |
| Q9CX30 | Protein YIF1B | Yif1b | P70451 | Tyrosine-protein kinase Fer | Fer | Q8BG67 | Protein EFR3 homolog A | Efr3a |
| O88522 | NF-kappa-B essential modulator | Ikbkg | Q8VI63 | MOB kinase activator 2 | Mob2 | Q8K2Q0 | COMM domain-containing protein 9 | Commd9 |
| Q9CY27 | Very-long-chain enoyl-CoA reductase | Tecr | P21956 | Lactadherin | Mfge8 | Q9WU00 | Nuclear respiratory factor 1 | Nrf1 |
| Q148V7 | RAB11-binding protein RELCH | Relch | Q8R313 | Exocyst complex component 6 | Exoc6 | Q8R1V4 | Transmembrane emp24 domain-containing protein 4 | Tmed4 |
| Q9JMH6 | Thioredoxin reductase 1, cytoplasmic | Txnrd1 | Q9CQ43 | Deoxyuridine 5-triphosphate nucleotidohydrolase | Dut | Q9QUI1 | Leucine repeat adapter protein 25 | Fam89b |
| Q8C3I8 | Protein HGH1 homolog | Hgh1 | Q64511 | DNA topoisomerase 2-beta | Top2b | Q99J93 | Interferon-induced transmembrane protein 2 | Ifitm2 |
| Q6PHU5 | Sortilin | Sort1 | Q4VBD2 | Transmembrane anterior posterior transformation protein 1 | Tapt1 | Q5F285 | Transmembrane protein 256 | Tmem256 |
| Q8R502 | Volume-regulated anion channel subunit LRRC8C | Lrrc8c | P30987 | Thromboxane A2 receptor | Tbxa2r | Q922B1 | ADP-ribose glycohydrolase MACROD1 | Macrod1 |
| Q8C0I1 | Alkyldihydroxyacetonephosphate synthase, peroxisomal | Agps | P17433 | Transcription factor PU.1 | Spi1 | Q99PQ1 | Tripartite motif-containing protein 12A | Trim12a |
| Q8JZR0 | Long-chain-fatty-acid--CoA ligase 5 | Acsl5 | P84309 | Adenylate cyclase type 5 | Adcy5 | P56379 | ATP synthase subunit ATP5MPL, mitochondrial | Atp5mpl |
| Q9DC29 | ATP-binding cassette sub-family B member 6 | Abcb6 | P63034 | Cytohesin-2 | Cyth2 | P61600 | N-alpha-acetyltransferase 20 | Naa20 |
| P0C191 | Platelet glycoprotein VI | Gp6 | E9Q6P5 | Tetratricopeptide repeat protein 7B | Ttc7b | Q9Z2R9 | Eukaryotic translation initiation factor 2-alpha kinase 1 | Eif2ak1 |
| P34152 | Focal adhesion kinase 1 | Ptk2 | Q8CCJ3 | E3 UFM1-protein ligase 1 | Ufl1 | Q8BJM7 | S-adenosyl-L-methionine-dependent tRNA 4-demethylwyosine synthase TYW1 | Tyw1 |
| Q9DBE8 | Alpha-1,3/1,6-mannosyltransferase ALG2 | Alg2 | Q9CQ56 | Vesicle transport protein USE1 | Use1 | P47758 | Signal recognition particle receptor subunit beta | Srprb |
| Q03350 | Thrombospondin-2 | Thbs2 | Q99M31 | Heat shock 70 kDa protein 14 | Hspa14 | O88983 | Syntaxin-8 | Stx8 |
| Q91WS0 | CDGSH iron-sulfur domain-containing protein 1 | Cisd1 | P97390 | Vacuolar protein sorting-associated protein 45 | Vps45 | Q9EQQ2 | Protein YIPF5 | Yipf5 |
| Q99L43 | Phosphatidate cytidylyltransferase 2 | Cds2 | Q8K1X4 | Nck-associated protein 1-like | Nckap1l | Q9QZ25 | Vascular non-inflammatory molecule 3 | Vnn3 |
| P36536 | GTP-binding protein SAR1a | Sar1a | Q68FH4 | N-acetylgalactosamine kinase | Galk2 | Q9ER88 | Small ribosomal subunit protein mS29 | Dap3 |
| Q4FJU9 | Transmembrane protein 40 | Tmem40 | Q8R5F3 | ADP-ribose glycohydrolase OARD1 | Oard1 | Q9CXW2 | Small ribosomal subunit protein mS22 | Mrps22 |
| Q99LR1 | Lysophosphatidylserine lipase ABHD12 | Abhd12 | A2AIV8 | Caspase recruitment domain-containing protein 9 | Card9 | Q8BTE6 | Protein Wfdc21 | Wfdc21 |
| Q9CQH3 | NADH dehydrogenase [ubiquinone] 1 beta subcomplex subunit 5, mitochondrial | Ndufb5 | Q9DCE5 | p21-activated protein kinase-interacting protein 1 | Pak1ip1 | Q2TBE6 | Phosphatidylinositol 4-kinase type 2-alpha | Pi4k2a |
| P70288 | Histone deacetylase 2 | Hdac2 | P03921 | NADH-ubiquinone oxidoreductase chain 5 | Mtnd5 | Q9CXR1 | Dehydrogenase/reductase SDR family member 7 | Dhrs7 |
| Q3T9M1 | Sphingosine-1-phosphate transporter MFSD2B | Mfsd2b | P97742 | Carnitine O-palmitoyltransferase 1, liver isoform | Cpt1a | Q3TIX9 | U4/U6.U5 tri-snRNP-associated protein 2 | Usp39 |
| Q9D6K8 | FUN14 domain-containing protein 2 | Fundc2 | Q8VD65 | Phosphoinositide 3-kinase regulatory subunit 4 | Pik3r4 | P32233 | Developmentally-regulated GTP-binding protein 1 | Drg1 |
| Q9Z1G4 | V-type proton ATPase 116 kDa subunit a 1 | Atp6v0a1 | P10404 | MLV-related proviral Env polyprotein | | Q8K2M0 | Large ribosomal subunit protein mL38 | Mrpl38 |
| A2RSJ4 | Bridge-like lipid transfer protein family member 3B | Bltp3b | Q9D0W5 | Peptidyl-prolyl cis-trans isomerase-like 1 | Ppil1 | Q91W96 | Anaphase-promoting complex subunit 4 | Anapc4 |
| O70378 | ER membrane protein complex subunit 8 | Emc8 | Q8VBW6 | NEDD8-activating enzyme E1 regulatory subunit | Nae1 | Q9CQ40 | Large ribosomal subunit protein mL49 | Mrpl49 |
| Q08879 | Fibulin-1 | Fbln1 | Q8VEH8 | Endoplasmic reticulum lectin 1 | Erlec1 | Q60928 | Glutathione hydrolase 1 proenzyme | Ggt1 |
| Q61387 | Cytochrome c oxidase subunit 7A-related protein, mitochondrial | Cox7a2l | Q8C6U2 | Solute carrier family 66 member 3 | Slc66a3 | Q9D0L7 | Armadillo repeat-containing protein 10 | Armc10 |
| Q9Z2D6 | Methyl-CpG-binding protein 2 | Mecp2 | B9EJ86 | Oxysterol-binding protein-related protein 8 | Osbpl8 | P27808 | Alpha-1,3-mannosyl-glycoprotein 2-beta-N-acetylglucosaminyltransferase | Mgat1 |
| Q8BPU7 | Engulfment and cell motility protein 1 | Elmo1 | Q91V01 | Lysophospholipid acyltransferase 5 | Lpcat3 | Q99K95 | Replication termination factor 2 | Rtf2 |
| Q9QZN4 | F-box only protein 6 | Fbxo6 | P28028 | Serine/threonine-protein kinase B-raf | Braf | Q9CR64 | Protein kish-A | Tmem167a |
| P35285 | Ras-related protein Rab-22A | Rab22a | Q6PGF7 | Exocyst complex component 8 | Exoc8 | P15392 | Cytochrome P450 2A4 | Cyp2a4 |
| Q8K2Z4 | Condensin complex subunit 1 | Ncapd2 | Q9CQA9 | Cancer-related nucleoside-triphosphatase homolog | Ntpcr | Q8BFP9 | [Pyruvate dehydrogenase (acetyl-transferring)] kinase isozyme 1, mitochondrial | Pdk1 |
| Q9CZB0 | Succinate dehydrogenase cytochrome b560 subunit, mitochondrial | Sdhc | Q9CQ71 | Replication protein A 14 kDa subunit | Rpa3 | Q924N4 | Solute carrier family 12 member 6 | Slc12a6 |
| Q8BHL3 | TBC1 domain family member 10B | Tbc1d10b | Q9CXV1 | Succinate dehydrogenase [ubiquinone] cytochrome b small subunit, mitochondrial | Sdhd | Q91Y57 | Sialic acid-binding Ig-like lectin 12 | Siglec12 |
| Q9CRB9 | MICOS complex subunit Mic19 | Chchd3 | P24604 | Tyrosine-protein kinase Tec | Tec | Q8BGK6 | Y+L amino acid transporter 2 | Slc7a6 |
| Q8BU33 | 2-hydroxyacyl-CoA lyase 2 | Ilvbl | Q3UUG6 | TBC1 domain family member 24 | Tbc1d24 | Q63961 | Endoglin | Eng |
| Q9Z2Z6 | Mitochondrial carnitine/acylcarnitine carrier protein | Slc25a20 | Q3UJP5 | Cilia- and flagella-associated protein 418 | Cfap418 | G3X9K3 | Brefeldin A-inhibited guanine nucleotide-exchange protein 1 | Arfgef1 |
| Q8R5J9 | PRA1 family protein 3 | Arl6ip5 | Q9D2V8 | Major facilitator superfamily domain-containing protein 10 | Mfsd10 | Q8VEH5 | EPM2A-interacting protein 1 | Epm2aip1 |
| Q9D8V7 | Signal peptidase complex catalytic subunit SEC11C | Sec11c | P58021 | Transmembrane 9 superfamily member 2 | Tm9sf2 | Q8BVD5 | MAGUK p55 subfamily member 7 | Mpp7 |
| Q80UU9 | Membrane-associated progesterone receptor component 2 | Pgrmc2 | Q6PA06 | Atlastin-2 | Atl2 | Q9JJ00 | Phospholipid scramblase 1 | Plscr1 |
| P55065 | Phospholipid transfer protein | Pltp | O70305 | Ataxin-2 | Atxn2 | Q9WV30 | Nuclear factor of activated T-cells 5 | Nfat5 |
| Q8CIV8 | Tubulin-specific chaperone E | Tbce | Q9R099 | Transducin beta-like protein 2 | Tbl2 | P33766 | fMet-Leu-Phe receptor | Fpr1 |
| P31750 | RAC-alpha serine/threonine-protein kinase | Akt1 | Q91XD7 | Protein disulfide isomerase Creld1 | Creld1 | Q8CFI7 | DNA-directed RNA polymerase II subunit RPB2 | Polr2b |
| Q8C7K6 | Prenylcysteine oxidase-like | Pcyox1l | P12791 | Cytochrome P450 2B10 | Cyp2b10 | Q8VE18 | Nonsense-mediated mRNA decay factor SMG8 | Smg8 |
| Q9CPQ3 | Mitochondrial import receptor subunit TOM22 homolog | Tomm22 | O08832 | Polypeptide N-acetylgalactosaminyltransferase 4 | Galnt4 | O55242 | Sigma non-opioid intracellular receptor 1 | Sigmar1 |
| P70295 | Lipid droplet-regulating VLDL assembly factor AUP1 | Aup1 | Q9DBL9 | 1-acylglycerol-3-phosphate O-acyltransferase ABHD5 | Abhd5 | Q80U58 | Pumilio homolog 2 | Pum2 |
| E9Q3L2 | Phosphatidylinositol 4-kinase alpha | Pi4ka | Q9WUQ2 | Prolactin regulatory element-binding protein | Preb | Q64287 | Interferon regulatory factor 4 | Irf4 |
| Q9Z2I0 | Mitochondrial proton/calcium exchanger protein | Letm1 | Q9EPQ7 | StAR-related lipid transfer protein 5 | Stard5 | E9Q236 | ATP-binding cassette sub-family C member 4 | Abcc4 |
| O88441 | Metaxin-2 | Mtx2 | Q91V04 | Translocating chain-associated membrane protein 1 | Tram1 | Q99PT9 | Kinesin-like protein KIF19 | Kif19 |
| P26369 | Splicing factor U2AF 65 kDa subunit | U2af2 | Q3TBW2 | Large ribosomal subunit protein uL10m | Mrpl10 | P35330 | Intercellular adhesion molecule 2 | Icam2 |
| Q9JHJ3 | Glycosylated lysosomal membrane protein | Glmp | O09111 | NADH dehydrogenase [ubiquinone] 1 beta subcomplex subunit 11, mitochondrial | Ndufb11 | Q9D0B0 | Serine/arginine-rich splicing factor 9 | Srsf9 |
| Q78IK4 | MICOS complex subunit Mic27 | Apool | Q80ZW2 | Protein THEM6 | Them6 | Q9D0G0 | Large ribosomal subunit protein mL65 | Mrps30 |
| O35609 | Secretory carrier-associated membrane protein 3 | Scamp3 | Q811J3 | Iron-responsive element-binding protein 2 | Ireb2 | Q9D8C2 | Tetraspanin-13 | Tspan13 |
| O88520 | Leucine-rich repeat protein SHOC-2 | Shoc2 | P70191 | TNF receptor-associated factor 5 | Traf5 | Q6P5U7 | NACHT and WD repeat domain-containing protein 2 | Nwd2 |
| O54950 | 5-AMP-activated protein kinase subunit gamma-1 | Prkag1 | P39061 | Collagen alpha-1(XVIII) chain | Col18a1 | Q80TL7 | Protein MON2 homolog | Mon2 |
| Q91WC9 | Diacylglycerol lipase-beta | Daglb | Q3UJD6 | Ubiquitin carboxyl-terminal hydrolase 19 | Usp19 | D3YV10 | Coiled-coil domain-containing protein 13 | Ccdc13 |
| P20491 | High affinity immunoglobulin epsilon receptor subunit gamma | Fcer1g | Q62193 | Replication protein A 32 kDa subunit | Rpa2 | Q8R1J9 | Torsin-2A | Tor2a |
| P48999 | Polyunsaturated fatty acid 5-lipoxygenase | Alox5 | Q6TEK5 | Vitamin K epoxide reductase complex subunit 1-like protein 1 | Vkorc1l1 | Q9JLZ6 | Hypermethylated in cancer 2 protein | Hic2 |
| P46978 | Dolichyl-diphosphooligosaccharide--protein glycosyltransferase subunit STT3A | Stt3a | O88597 | Beclin-1 | Becn1 | Q9QZD8 | Mitochondrial dicarboxylate carrier | Slc25a10 |
| D3YZP9 | Coiled-coil domain-containing protein 6 | Ccdc6 | Q8BK72 | Small ribosomal subunit protein mS27 | Mrps27 | Q9D1X9 | Transmembrane protein 50B | Tmem50b |
| Q8CHK3 | Lysophospholipid acyltransferase 7 | Mboat7 | Q8C996 | Transmembrane protein 163 | Tmem163 | Q9D9G2 | Phosphatidylethanolamine-binding protein 4 | Pebp4 |
| Q9CPU4 | Glutathione S-transferase 3, mitochondrial | Mgst3 | Q8BG93 | Nucleotide triphosphate diphosphatase NUDT15 | Nudt15 | Q8CF94 | Blood group Rh(D) polypeptide | Rhd |
| O88738 | Baculoviral IAP repeat-containing protein 6 | Birc6 | O89013 | Leptin receptor gene-related protein | Leprot | Q91W89 | Alpha-mannosidase 2C1 | Man2c1 |
| P47934 | Carnitine O-acetyltransferase | Crat | Q8CH77 | Neuron navigator 1 | Nav1 | Q8BJL1 | F-box only protein 30 | Fbxo30 |
| Q62241 | U1 small nuclear ribonucleoprotein C | Snrpc | Q3TDD9 | Protein phosphatase 1 regulatory subunit 21 | Ppp1r21 | Q8VCS3 | Glycosaminoglycan xylosylkinase | Fam20b |
| Q61211 | Eukaryotic translation initiation factor 2D | Eif2d | Q9WV96 | Mitochondrial import inner membrane translocase subunit Tim10 B | Timm10b | Q9D3S3 | Sorting nexin-29 | Snx29 |
| Q9D8S3 | ADP-ribosylation factor GTPase-activating protein 3 | Arfgap3 | Q9EQI8 | Large ribosomal subunit protein mL46 | Mrpl46 | Q8R242 | Di-N-acetylchitobiase | Ctbs |
| Q8QZY6 | Tetraspanin-14 | Tspan14 | Q91WM2 | Haloacid dehalogenase-like hydrolase domain-containing 5 | Hdhd5 | O08848 | RNA-binding protein Ro60 | RO60 |
| Q7TMF3 | NADH dehydrogenase [ubiquinone] 1 alpha subcomplex subunit 12 | Ndufa12 | Q6A068 | Cell division cycle 5-like protein | Cdc5l | Q9CQK7 | RWD domain-containing protein 1 | Rwdd1 |
| P35283 | Ras-related protein Rab-12 | Rab12 | Q80XN0 | D-beta-hydroxybutyrate dehydrogenase, mitochondrial | Bdh1 | P61166 | Transmembrane protein 258 | Tmem258 |
| P25976 | Nucleolar transcription factor 1 | Ubtf | Q505B7 | Protein archease | Zbtb8os | Q99KI3 | ER membrane protein complex subunit 3 | Emc3 |
| Q80Y98 | Phospholipase DDHD2 | Ddhd2 | Q8BTY8 | Sec1 family domain-containing protein 2 | Scfd2 | Q9CQF0 | Large ribosomal subunit protein uL11m | Mrpl11 |
| Q91VM3 | WD repeat domain phosphoinositide-interacting protein 4 | Wdr45 | P0DN34 | NADH dehydrogenase [ubiquinone] 1 beta subcomplex subunit 1 | Ndufb1 | Q9WVH9 | Fibulin-5 | Fbln5 |
| Q99JB2 | Stomatin-like protein 2, mitochondrial | Stoml2 | O54692 | Centromere/kinetochore protein zw10 homolog | Zw10 | Q80TA9 | Ectopic P granules protein 5 homolog | Epg5 |
| Q9CR67 | Transmembrane protein 33 | Tmem33 | Q9CPU2 | NADH dehydrogenase [ubiquinone] 1 beta subcomplex subunit 2, mitochondrial | Ndufb2 | P26187 | Methylated-DNA--protein-cysteine methyltransferase | Mgmt |
| Q9D6J5 | NADH dehydrogenase [ubiquinone] 1 beta subcomplex subunit 8, mitochondrial | Ndufb8 | Q8VEK0 | Cell cycle control protein 50A | Tmem30a | Q8VCS0 | N-acetylmuramoyl-L-alanine amidase | Pglyrp2 |
| Q9R008 | Mevalonate kinase | Mvk | Q922Q1 | Mitochondrial amidoxime reducing component 2 | Mtarc2 | Q8R0F8 | Acylpyruvase FAHD1, mitochondrial | Fahd1 |
| Q6ZPS6 | Ankyrin repeat and IBR domain-containing protein 1 | Ankib1 | Q64310 | Surfeit locus protein 4 | Surf4 | Q8C0M9 | Isoaspartyl peptidase/L-asparaginase | Asrgl1 |
| P08607 | C4b-binding protein | C4bpa | Q811S7 | Upstream-binding protein 1 | Ubp1 | Q9D4H1 | Exocyst complex component 2 | Exoc2 |
| Q8BX80 | Cytosolic endo-beta-N-acetylglucosaminidase | Engase | P62746 | Rho-related GTP-binding protein RhoB | Rhob | P99028 | Cytochrome b-c1 complex subunit 6, mitochondrial | Uqcrh |
| O70293 | G protein-coupled receptor kinase 6 | Grk6 | Q9R0Q9 | Mannose-P-dolichol utilization defect 1 protein | Mpdu1 | Q9D1L9 | Ragulator complex protein LAMTOR5 | Lamtor5 |

**Supplementary Table S5 The information of proteins highly expressed in leukocytes of the CPAP group.**

| **Protein** | **Description** | **Gene** | **Protein** | **Description** | **Gene** | | **Protein** | **Description** | **Gene** |
| --- | --- | --- | --- | --- | --- | --- | --- | --- | --- |
| Q3UDK1 | TRAF-type zinc finger domain-containing protein 1 | Trafd1 | Q9QXL1 | Kinesin-like protein KIF21B | | Kif21b | O35900 | U6 snRNA-associated Sm-like protein LSm2 | Lsm2 |
| Q9JL26 | Formin-like protein 1 | Fmnl1 | O35309 | N-myc-interactor | | Nmi | P70274 | Selenoprotein P | Selenop |
| Q6NSR8 | Probable aminopeptidase NPEPL1 | Npepl1 | Q8K182 | Complement component C8 alpha chain | | C8a | Q8VC30 | Triokinase/FMN cyclase | Tkfc |
| O88712 | C-terminal-binding protein 1 | Ctbp1 | P09813 | Apolipoprotein A-II | | Apoa2 | Q01279 | Epidermal growth factor receptor | Egfr |
| P51855 | Glutathione synthetase | Gss | P07759 | Serine protease inhibitor A3K | | Serpina3k | Q8R4X3 | RNA-binding protein 12 | Rbm12 |
| P28665 | Murinoglobulin-1 | Mug1 | Q3UZA1 | CapZ-interacting protein | | Rcsd1 | P51125 | Calpastatin | Cast |
| O70475 | UDP-glucose 6-dehydrogenase | Ugdh | P33622 | Apolipoprotein C-III | | Apoc3 | Q9JLI6 | Selenocysteine lyase | Scly |
| P50543 | Protein S100-A11 | S100a11 | O35143 | ATPase inhibitor, mitochondrial | | Atp5if1 | P07091 | Protein S100-A4 | S100a4 |
| P97492 | Regulator of G-protein signaling 14 | Rgs14 | Q3U5Q7 | UMP-CMP kinase 2, mitochondrial | | Cmpk2 | P01831 | Thy-1 membrane glycoprotein | Thy1 |
| Q9D6Y7 | Mitochondrial peptide methionine sulfoxide reductase | Msra | P56382 | ATP synthase subunit epsilon, mitochondrial | | Atp5f1e | P0DOV1 | Interferon-activable protein 205-B | Mnda |
| Q3U0V1 | Far upstream element-binding protein 2 | Khsrp | Q9D8C4 | Interferon-induced 35 kDa protein homolog | | Ifi35 | P14430 | H-2 class I histocompatibility antigen, Q8 alpha chain | H2-Q8 |
| O54988 | STE20-like serine/threonine-protein kinase | Slk | Q8K003 | Translation machinery-associated protein 7 | | Tma7 | Q9EQN3 | TSC22 domain family protein 4 | Tsc22d4 |
| Q9EST5 | Acidic leucine-rich nuclear phosphoprotein 32 family member B | Anp32b | P27870 | Proto-oncogene vav | | Vav1 | Q99PG2 | Opioid growth factor receptor | Ogfr |
| P63166 | Small ubiquitin-related modifier 1 | Sumo1 | B7ZNG4 | Tastin | | Troap | Q9CQ54 | NADH dehydrogenase [ubiquinone] 1 subunit C2 | Ndufc2 |
| P42209 | Septin-1 | Septin1 | P22599 | Alpha-1-antitrypsin 1-2 | | Serpina1b | Q6ZWY8 | Thymosin beta-10 | Tmsb10 |
| Q91ZJ5 | UTP--glucose-1-phosphate uridylyltransferase | Ugp2 | P42703 | Leukemia inhibitory factor receptor | | Lifr | P68404 | Protein kinase C beta type | Prkcb |
| Q8BH35 | Complement component C8 beta chain | C8b | Q3THS6 | S-adenosylmethionine synthase isoform type-2 | | Mat2a | Q61029 | Lamina-associated polypeptide 2, isoforms beta/delta/epsilon/gamma | Tmpo |
| Q9CSU0 | Regulation of nuclear pre-mRNA domain-containing protein 1B | Rprd1b | Q00896 | Alpha-1-antitrypsin 1-3 | | Serpina1c | P28076 | Proteasome subunit beta type-9 | Psmb9 |
| Q9D7I5 | Phospholysine phosphohistidine inorganic pyrophosphate phosphatase | Lhpp | Q3T9E4 | T-cell-specific guanine nucleotide triphosphate-binding protein 2 | | Tgtp2 | Q60821 | Zinc finger and BTB domain-containing protein 17 | Zbtb17 |
| P49290 | Eosinophil peroxidase | Epx | Q00519 | Xanthine dehydrogenase/oxidase | | Xdh | Q6ZWZ2 | Ubiquitin-conjugating enzyme E2 R2 | Ube2r2 |
| P02088 | Hemoglobin subunit beta-1 | Hbb-b1 | Q9Z0I7 | Schlafen family member 1 | | Slfn1 | Q9Z1F9 | SUMO-activating enzyme subunit 2 | Uba2 |
| P62257 | Ubiquitin-conjugating enzyme E2 H | Ube2h | Q64282 | Interferon-induced protein with tetratricopeptide repeats 1 | | Ifit1 | Q00623 | Apolipoprotein A-I | Apoa1 |
| Q9EPB4 | Apoptosis-associated speck-like protein containing a CARD | Pycard | Q64GA5 | Cytosolic phospholipase A2 gamma | | Pla2g4c | P01644 | Ig kappa chain V-V region HP R16.7 | |
| Q99JY3 | GTPase IMAP family member 4 | Gimap4 | P68134 | Actin, alpha skeletal muscle | | Acta1 | O70493 | Sorting nexin-12 | Snx12 |
| P97822 | Acidic leucine-rich nuclear phosphoprotein 32 family member E | Anp32e | Q9R088 | Thymidine kinase 2, mitochondrial | | Tk2 | Q8C3J5 | Dedicator of cytokinesis protein 2 | Dock2 |
| P62313 | U6 snRNA-associated Sm-like protein LSm6 | Lsm6 | Q9EPL0 | Xylosyltransferase 2 | | Xylt2 | P27546 | Microtubule-associated protein 4 | Map4 |
| O08784 | Treacle protein | Tcof1 | Q9CQI3 | Glia maturation factor beta | | Gmfb | P46664 | Adenylosuccinate synthetase isozyme 2 | Adss2 |
| Q8VI75 | Importin-4 | Ipo4 | P01898 | H-2 class I histocompatibility antigen, Q10 alpha chain | | H2-Q10 | Q3TMH2 | Secernin-3 | Scrn3 |
| Q64520 | Guanylate kinase | Guk1 | Q9D6Y9 | 1,4-alpha-glucan-branching enzyme | | Gbe1 | P08030 | Adenine phosphoribosyltransferase | Aprt |
| P50429 | Arylsulfatase B | Arsb | Q9CPT3 | N-acylneuraminate-9-phosphatase | | Nanp | P03953 | Complement factor D | Cfd |
| P31786 | Acyl-CoA-binding protein | Dbi | P43430 | Mast cell protease 8 | | Mcpt8 | P34928 | Apolipoprotein C-I | Apoc1 |
| Q9QWK4 | CD5 antigen-like | Cd5l | P11031 | Activated RNA polymerase II transcriptional coactivator p15 | | Sub1 | P04444 | Hemoglobin subunit beta-H1 | Hbb-bh1 |
| P02089 | Hemoglobin subunit beta-2 | Hbb-b2 | P16125 | L-lactate dehydrogenase B chain | | Ldhb | P06802 | Ectonucleotide pyrophosphatase/phosphodiesterase family member 1 | Enpp1 |
| P09528 | Ferritin heavy chain | Fth1 | Q8CCS6 | Polyadenylate-binding protein 2 | | Pabpn1 | Q8VCA8 | Secernin-2 | Scrn2 |
| Q60710 | Deoxynucleoside triphosphate triphosphohydrolase SAMHD1 | Samhd1 | Q64471 | Glutathione S-transferase theta-1 | | Gstt1 | P13439 | Uridine 5-monophosphate synthase | Umps |
| P01942 | Hemoglobin subunit alpha | Hba | P70429 | Ena/VASP-like protein | | Evl | P14824 | Annexin A6 | Anxa6 |
| Q9D1J3 | SAP domain-containing ribonucleoprotein | Sarnp | Q9D5V6 | Synapse-associated protein 1 | | Syap1 | P29391 | Ferritin light chain 1 | Ftl1 |
| Q922U1 | U4/U6 small nuclear ribonucleoprotein Prp3 | Prpf3 | Q61189 | Methylosome subunit pICln | | Clns1a | P97310 | DNA replication licensing factor MCM2 | Mcm2 |
| Q3TGF2 | Protein FAM107B | Fam107b | Q9QZB7 | Actin-related protein 10 | | Actr10 | Q9D554 | Splicing factor 3A subunit 3 | Sf3a3 |
| P23492 | Purine nucleoside phosphorylase | Pnp | O70370 | Cathepsin S | | Ctss | Q3U7U3 | F-box only protein 7 | Fbxo7 |
| O89020 | Afamin | Afm | P54227 | Stathmin | | Stmn1 | P17892 | Pancreatic lipase-related protein 2 | Pnliprp2 |
| Q8VCG4 | Complement component C8 gamma chain | C8g | P46414 | Cyclin-dependent kinase inhibitor 1B | | Cdkn1b | P00920 | Carbonic anhydrase 2 | Ca2 |
| P47739 | Aldehyde dehydrogenase, dimeric NADP-preferring | Aldh3a1 | Q61074 | Protein phosphatase 1G | | Ppm1g | Q99NE5 | Regulating synaptic membrane exocytosis protein 1 | Rims1 |
| P11352 | Glutathione peroxidase 1 | Gpx1 | Q64112 | Interferon-induced protein with tetratricopeptide repeats 2 | | Ifit2 | P31725 | Protein S100-A9 | S100a9 |
| Q9DAR7 | m7GpppX diphosphatase | Dcps | P01807 | Ig heavy chain V region X44 | | | P43346 | Deoxycytidine kinase | Dck |
| Q99MD9 | Nuclear autoantigenic sperm protein | Nasp | P51885 | Lumican | | Lum | P27005 | Protein S100-A8 | S100a8 |
| P07309 | Transthyretin | Ttr | Q60953 | Protein PML | | Pml | Q6WVG3 | BTB/POZ domain-containing protein KCTD12 | Kctd12 |
| P28650 | Adenylosuccinate synthetase isozyme 1 | Adss1 | Q9WVJ3 | Carboxypeptidase Q | | Cpq | Q9D7Q1 | Chitotriosidase-1 | Chit1 |
| Q9EQU5 | Protein SET | Set | Q8BR07 | Protein bicaudal D homolog 1 | | Bicd1 | Q9R1T4 | Septin-6 | Septin6 |
| Q91VF2 | Histamine N-methyltransferase | Hnmt | P04117 | Fatty acid-binding protein, adipocyte | | Fabp4 | Q8R326 | Paraspeckle component 1 | Pspc1 |
| G5E8P0 | Gamma-tubulin complex component 6 | Tubgcp6 | Q9QY24 | Z-DNA-binding protein 1 | | Zbp1 | P13634 | Carbonic anhydrase 1 | Ca1 |
| Q8R1S0 | Ubiquinone biosynthesis monooxygenase COQ6, mitochondrial | Coq6 | Q921E2 | Ras-related protein Rab-31 | | Rab31 | P23953 | Carboxylesterase 1C | Ces1c |
| Q91XB0 | Three-prime repair exonuclease 1 | Trex1 | Q3TTY5 | Keratin, type II cytoskeletal 2 epidermal | | Krt2 | Q5SVL6 | Rap1 GTPase-activating protein 2 | Rap1gap2 |
| Q68ED7 | CREB-regulated transcription coactivator 1 | Crtc1 | P01631 | Ig kappa chain V-II region 26-10 | |  | Q80XU3 | Nuclear ubiquitous casein and cyclin-dependent kinase substrate 1 | Nucks1 |
| Q91W39 | Nuclear receptor coactivator 5 | Ncoa5 | Q9D114 | Guanosine-3,5-bis(diphosphate) 3-pyrophosphohydrolase MESH1 | | Hddc3 | Q9Z0H7 | B-cell lymphoma/leukemia 10 | Bcl10 |
| Q5DU31 | Interactor protein for cytohesin exchange factors 1 | Ipcef1 | B2RY56 | RNA-binding protein 25 | | Rbm25 | P03976 | Ig kappa chain V-II region 17S29.1 |  |
| Q793I8 | TRAF-interacting protein with FHA domain-containing protein A | Tifa | Q6ZWM4 | U6 snRNA-associated Sm-like protein LSm8 | | Lsm8 | Q9DBR1 | 5-3 exoribonuclease 2 | Xrn2 |
| Q8CDA1 | Phosphatidylinositide phosphatase SAC2 | Inpp5f | Q64191 | N(4)-(beta-N-acetylglucosaminyl)-L-asparaginase | | Aga | P04938 | Major urinary protein 11 | Mup11 |
| Q80ZM7 | Transcription initiation factor IIA subunit 2 | Gtf2a2 | Q64345 | Interferon-induced protein with tetratricopeptide repeats 3 | | Ifit3 | Q8BVG4 | Dipeptidyl peptidase 9 | Dpp9 |
| O54786 | DNA fragmentation factor subunit alpha | Dffa | Q64261 | Cyclin-dependent kinase 6 | | Cdk6 | Q9JHS9 | Spliceosome-associated protein CWC15 homolog | Cwc15 |
| P58466 | Carboxy-terminal domain RNA polymerase II polypeptide A small phosphatase 1 | Ctdsp1 | Q3V3Q7 | Phosphofurin acidic cluster sorting protein 2 | | Pacs2 | Q3SXD3 | 5-deoxynucleotidase HDDC2 | Hddc2 |
| Q03717 | Potassium voltage-gated channel subfamily B member 1 | Kcnb1 | Q6P5G6 | UBX domain-containing protein 7 | | Ubxn7 | O89090 | Transcription factor Sp1 | Sp1 |
| Q3UFY7 | 7-methylguanosine phosphate-specific 5-nucleotidase | Nt5c3b | P18524 | Ig heavy chain V region RF | |  | B1AXH1 | NHS-like protein 2 | Nhsl2 |
| Q61400 | Carcinoembryonic antigen-related cell adhesion molecule 10 | Ceacam10 | Q6IR34 | G-protein-signaling modulator 1 | | Gpsm1 | Q9QYG0 | Protein NDRG2 | Ndrg2 |
| Q5DU57 | Spermatogenesis-associated protein 13 | Spata13 | Q5BLK4 | Terminal uridylyltransferase 7 | | Tut7 | Q8BFS6 | Serine/threonine-protein phosphatase CPPED1 | Cpped1 |
| G5E870 | E3 ubiquitin-protein ligase TRIP12 | Trip12 | P14847 | C-reactive protein | | Crp | P0CG14 | Decreased expression in renal and prostate cancer protein | Derpc |
| Q99N91 | Large ribosomal subunit protein bL34m | Mrpl34 | Q07968 | Coagulation factor XIII B chain | | F13b | Q99LL3 | Carbohydrate sulfotransferase 12 | Chst12 |
| O88199 | Carbohydrate sulfotransferase 3 | Chst3 | Q9QXG2 | Rab proteins geranylgeranyltransferase component A 1 | | Chm | Q9Z1R3 | Apolipoprotein M | Apom |
| Q9CV28 | Ubiquitin carboxyl-terminal hydrolase MINDY-3 | Mindy3 | Q99NF2 | NMDA receptor synaptonuclear signaling and neuronal migration factor | | Nsmf | Q8BGC4 | Prostaglandin reductase-3 | Ptgr3 |
| Q80XC2 | tRNA (adenine(58)-N(1))-methyltransferase catalytic subunit TRMT61A | Trmt61a | P63137 | Gamma-aminobutyric acid receptor subunit beta-2 | | Gabrb2 | Q5ND28 | Scavenger receptor class F member 1 | Scarf1 |
| Q9CXG3 | Peptidyl-prolyl cis-trans isomerase-like 4 | Ppil4 | P42859 | Huntingtin | | Htt |  |  |  |

**Supplementary Table S6 The information of proteins highly expressed in tumors of the model group.**

| **Protein** | **Description** | **Gene** | **Protein** | **Description** | **Gene** | **Protein** | **Description** | **Gene** |
| --- | --- | --- | --- | --- | --- | --- | --- | --- |
| Q64277 | ADP-ribosyl cyclase/cyclic ADP-ribose hydrolase 2 | Bst1 | P42128 | Forkhead box protein K1 | Foxk1 | Q3V3R4 | Integrin alpha-1 | Itga1 |
| P35288 | Ras-related protein Rab-23 | Rab23 | Q99MU3 | Double-stranded RNA-specific adenosine deaminase | Adar | A0A140LIF8 | Immunity-related GTPase family M protein 2 | Irgm2 |
| Q8R4H9 | Proton-coupled zinc antiporter SLC30A5 | Slc30a5 | Q8BUV3 | Gephyrin | Gphn | B1AY13 | Ubiquitin carboxyl-terminal hydrolase 24 | Usp24 |
| Q8BYA0 | Tubulin-specific chaperone D | Tbcd | Q9D7M1 | Glucose-induced degradation protein 8 homolog | Gid8 | Q3U1Y4 | DENN domain-containing protein 4B | Dennd4b |
| P10518 | Delta-aminolevulinic acid dehydratase | Alad | A2ADY9 | Protein DDI1 homolog 2 | Ddi2 | Q91WG2 | Rab GTPase-binding effector protein 2 | Rabep2 |
| Q9JHP7 | Protein O-glucosyltransferase 2 | Poglut2 | Q9JL26 | Formin-like protein 1 | Fmnl1 | P70158 | Acid sphingomyelinase-like phosphodiesterase 3a | Smpdl3a |
| Q8BHA3 | D-aminoacyl-tRNA deacylase 2 | Dtd2 | Q9D4J7 | PHD finger protein 6 | Phf6 | Q8VD66 | (Lyso)-N-acylphosphatidylethanolamine lipase | Abhd4 |
| Q8BTI9 | Phosphatidylinositol 4,5-bisphosphate 3-kinase catalytic subunit beta isoform | Pik3cb | Q6KAR6 | Exocyst complex component 3 | Exoc3 | Q9CQL0 | Protein N-lysine methyltransferase METTL21A | Mettl21A |
| O70579 | Peroxisomal membrane protein PMP34 | Slc25a17 | Q6NZQ2 | Probable ATP-dependent RNA helicase DDX31 | Ddx31 | Q8VDQ1 | Prostaglandin reductase 2 | Ptgr2 |
| P27870 | Proto-oncogene vav | Vav1 | P52431 | DNA polymerase delta catalytic subunit | Pold1 | O88879 | Apoptotic protease-activating factor 1 | Apaf1 |
| Q02105 | Complement C1q subcomponent subunit C | C1qc | P08226 | Apolipoprotein E | Apoe | Q8VCB1 | Nucleoporin NDC1 | Ndc1 |
| Q60590 | Alpha-1-acid glycoprotein 1 | Orm1 | Q99LI9 | Polyribonucleotide 5-hydroxyl-kinase Clp1 | Clp1 | P97313 | DNA-dependent protein kinase catalytic subunit | Prkdc |
| Q80XU3 | Nuclear ubiquitous casein and cyclin-dependent kinase substrate 1 | Nucks1 | P50172 | 11-beta-hydroxysteroid dehydrogenase 1 | Hsd11b1 | Q69ZB8 | Zinc finger CCHC domain-containing protein 2 | Zcchc2 |
| Q148V8 | Protein FAM83H | Fam83h | Q9DB10 | Essential MCU regulator, mitochondrial | Smdt1 | Q9Z210 | Peroxisomal membrane protein 11B | Pex11b |
| O08582 | GTP-binding protein 1 | Gtpbp1 | P29477 | Nitric oxide synthase, inducible | Nos2 | P06537 | Glucocorticoid receptor | Nr3c1 |
| Q8K4P0 | pre-mRNA 3 end processing protein WDR33 | Wdr33 | Q9DB25 | Dolichyl-phosphate beta-glucosyltransferase | Alg5 | Q99PT9 | Kinesin-like protein KIF19 | Kif19 |
| Q91WP6 | Serine protease inhibitor A3N | Serpina3n | Q60847 | Collagen alpha-1(XII) chain | Col12a1 | Q80Y56 | Rabenosyn-5 | Rbsn |
| Q3UJB9 | Enhancer of mRNA-decapping protein 4 | Edc4 | Q8BIK4 | Dedicator of cytokinesis protein 9 | Dock9 | Q6PAM1 | Alpha-taxilin | Txlna |
| Q8BMA6 | Signal recognition particle subunit SRP68 | Srp68 | Q80UZ2 | Protein SDA1 homolog | Sdad1 | Q80UP3 | Diacylglycerol kinase zeta | Dgkz |
| P97789 | 5-3 exoribonuclease 1 | Xrn1 | O88520 | Leucine-rich repeat protein SHOC-2 | Shoc2 | Q3U962 | Collagen alpha-2(V) chain | Col5a2 |
| Q8CI94 | Glycogen phosphorylase, brain form | Pygb | P23249 | Putative helicase MOV-10 | Mov10 | B9EJ80 | PDZ domain-containing protein 8 | Pdzd8 |
| P97369 | Neutrophil cytosol factor 4 | Ncf4 | P10810 | Monocyte differentiation antigen CD14 | Cd14 | P06342 | H-2 class II histocompatibility antigen, A-Q beta chain | H2-Ab1 |
| A2AAY5 | SH3 and PX domain-containing protein 2B | Sh3pxd2b | Q0VGB7 | Serine/threonine-protein phosphatase 4 regulatory subunit 2 | Ppp4r2 | Q9DB42 | Zinc finger protein 593 | Znf593 |
| P32233 | Developmentally-regulated GTP-binding protein 1 | Drg1 | P05367 | Serum amyloid A-2 protein | Saa2 | Q62192 | CD180 antigen | Cd180 |
| Q9JK30 | Origin recognition complex subunit 3 | Orc3 | Q9JHK4 | Geranylgeranyl transferase type-2 subunit alpha | Rabggta | A2A791 | Zinc finger MYM-type protein 4 | Zmym4 |
| E9PZJ8 | Activating signal cointegrator 1 complex subunit 3 | Ascc3 | Q8BTY8 | Sec1 family domain-containing protein 2 | Scfd2 | Q7TQC5 | Aprataxin | Aptx |
| Q9EQ06 | Estradiol 17-beta-dehydrogenase 11 | Hsd17b11 | Q571I9 | Aldehyde dehydrogenase family 16 member A1 | Aldh16a1 | Q9QY06 | Unconventional myosin-IXb | Myo9b |
| Q9WTX6 | Cullin-1 | Cul1 | Q9Z0E0 | Neurochondrin | Ncdn | P70315 | Actin nucleation-promoting factor WAS | Was |
| P48722 | Heat shock 70 kDa protein 4L | Hspa4l | A8C756 | Thyroid adenoma-associated protein homolog | Thada | P29387 | Guanine nucleotide-binding protein subunit beta-4 | Gnb4 |
| Q6PAR5 | GTPase-activating protein and VPS9 domain-containing protein 1 | Gapvd1 | P48999 | Polyunsaturated fatty acid 5-lipoxygenase | Alox5 | Q9QWV9 | Cyclin-T1 | Ccnt1 |
| Q8VD65 | Phosphoinositide 3-kinase regulatory subunit 4 | Pik3r4 | P97772 | Metabotropic glutamate receptor 1 | Grm1 | Q8QZV7 | Integrator complex subunit 13 | IntS13 |
| Q7TT37 | Elongator complex protein 1 | Elp1 | Q64324 | Syntaxin-binding protein 2 | Stxbp2 | Q9WTS2 | Alpha-(1,6)-fucosyltransferase | Fut8 |
| Q8BH24 | Transmembrane 9 superfamily member 4 | Tm9sf4 | P59729 | Ras and Rab interactor 3 | Rin3 | Q9D7A8 | Armadillo repeat-containing protein 1 | Armc1 |
| Q91Z67 | SLIT-ROBO Rho GTPase-activating protein 2 | Srgap2 | P70248 | Unconventional myosin-If | Myo1f | Q8K337 | Type II inositol 1,4,5-trisphosphate 5-phosphatase | Inpp5b |
| Q7TMY7 | Importin-8 | Ipo8 | E9Q414 | Apolipoprotein B-100 | Apob | Q3TMH2 | Secernin-3 | Scrn3 |
| B2RY04 | Dedicator of cytokinesis protein 5 | Dock5 | Q6PF93 | Phosphatidylinositol 3-kinase catalytic subunit type 3 | Pik3c3 | Q9CS00 | Splicing factor Cactin | Cactin |
| Q9D2V8 | Major facilitator superfamily domain-containing protein 10 | Mfsd10 | Q9JJK2 | LanC-like protein 2 | Lancl2 | Q6PCM1 | Lysine-specific demethylase 3A | Kdm3a |
| Q8VC88 | Grancalcin | Gca | Q9Z1G4 | V-type proton ATPase 116 kDa subunit a 1 | Atp6v0a1 | Q9QZR0 | E3 ubiquitin-protein ligase RNF25 | Rnf25 |
| Q9JHH6 | Carboxypeptidase B2 | Cpb2 | Q9CXP4 | Rho GTPase-activating protein 8 | Arhgap8 | Q3TC46 | Protein PAT1 homolog 1 | Patl1 |
| P49615 | Cyclin-dependent kinase 5 | Cdk5 | P17897 | Lysozyme C-1 | Lyz1 | Q8CCN5 | BCAS3 microtubule associated cell migration factor | Bcas3 |
| Q8CI95 | Oxysterol-binding protein-related protein 11 | Osbpl11 | P35174 | Stefin-2 | Stfa2 | Q9JI44 | DNA methyltransferase 1-associated protein 1 | Dmap1 |
| Q9D8U6 | Mast cell-expressed membrane protein 1 | Mcemp1 | Q05921 | 2-5A-dependent ribonuclease | Rnasel | Q8VDM1 | Zinc finger CCCH-type with G patch domain-containing protein | Zgpat |
| Q62383 | Transcription elongation factor SPT6 | Supt6h | P05366 | Serum amyloid A-1 protein | Saa1 | O35963 | Ras-related protein Rab-33B | Rab33b |
| A2AT37 | Regulator of nonsense transcripts 2 | Upf2 | Q925S4 | Interleukin-24 | Il24 | Q6ZPR5 | Sphingomyelin phosphodiesterase 4 | Smpd4 |
| Q8BX57 | PX domain-containing protein kinase-like protein | Pxk | Q8BTT6 | U3 small nucleolar RNA-associated protein 25 homolog | Utp25 | Q921I6 | SH3 domain-binding protein 4 | Sh3bp4 |
| Q8R2Y0 | Monoacylglycerol lipase ABHD6 | Abhd6 | Q9Z2V5 | Histone deacetylase 6 | Hdac6 | Q99P69 | Kinetochore protein Nuf2 | Nuf2 |
| Q80YV3 | Transformation/transcription domain-associated protein | Trrap | P28862 | Stromelysin-1 | Mmp3 | Q8BG48 | Serine/threonine-protein kinase 17B | Stk17b |
| P12246 | Serum amyloid P-component | Apcs | Q6ZQH8 | Nucleoporin NUP188 | Nup188 | P49135 | General transcription and DNA repair factor IIH helicase subunit XPB | Ercc3 |
| Q9QY81 | Nuclear pore membrane glycoprotein 210 | Nup210 | B9EJ86 | Oxysterol-binding protein-related protein 8 | Osbpl8 | A6X919 | Probable C-mannosyltransferase DPY19L1 | Dpy19l1 |
| Q9EQF2 | Kell blood group glycoprotein homolog | Kel | P97447 | Four and a half LIM domains protein 1 | Fhl1 | Q9EP89 | Serine beta-lactamase-like protein LACTB, mitochondrial | Lactb |
| Q9ERA6 | Tuftelin-interacting protein 11 | Tfip11 | P23927 | Alpha-crystallin B chain | Cryab | P48377 | MHC class II regulatory factor RFX1 | Rfx1 |
| Q8C147 | Dedicator of cytokinesis protein 8 | Dock8 | Q8BQZ4 | Ral GTPase-activating protein subunit beta | Ralgapb | Q61333 | Tumor necrosis factor alpha-induced protein 2 | Tnfaip2 |
| Q61093 | Cytochrome b-245 heavy chain | Cybb | Q6P2K6 | Serine/threonine-protein phosphatase 4 regulatory subunit 3A | Ppp4r3a | Q6Y5D8 | Rho GTPase-activating protein 10 | Arhgap10 |
| Q8VI93 | 2-5-oligoadenylate synthase 3 | Oas3 | E9Q3L2 | Phosphatidylinositol 4-kinase alpha | Pi4ka | Q9CY57 | Chromatin target of PRMT1 protein | Chtop |
| Q3UXZ6 | Protein FAM81A | Fam81a | Q99LD4 | COP9 signalosome complex subunit 1 | Gps1 | Q9D720 | Non-structural maintenance of chromosomes element 1 homolog | Nsmce1 |
| Q01149 | Collagen alpha-2(I) chain | Col1a2 | Q9ESJ0 | Exportin-4 | Xpo4 | A2ALW5 | DnaJ homolog subfamily C member 25 | Dnajc25 |
| Q80U58 | Pumilio homolog 2 | Pum2 | Q9ESC8 | AF4/FMR2 family member 4 | Aff4 | Q8K1X4 | Nck-associated protein 1-like | Nckap1l |
| P17183 | Gamma-enolase | Eno2 | P07361 | Alpha-1-acid glycoprotein 2 | Orm2 | P97868 | E3 ubiquitin-protein ligase RBBP6 | Rbbp6 |
| P22682 | E3 ubiquitin-protein ligase CBL | Cbl | Q3U2P1 | Protein transport protein Sec24A | Sec24a | O89103 | Complement component C1q receptor | Cd93 |
| Q924W5 | Structural maintenance of chromosomes protein 6 | Smc6 | P12804 | Fibroleukin | Fgl2 | Q8K2I1 | Protein farnesyltransferase subunit beta | Fntb |
| Q60766 | Immunity-related GTPase family M protein 1 | Irgm1 | Q9DCC8 | Mitochondrial import receptor subunit TOM20 homolog | Tomm20 | P35761 | Dual specificity protein kinase TTK | Ttk |
| Q9ER69 | Pre-mRNA-splicing regulator WTAP | Wtap | Q3V1L4 | Cytosolic purine 5-nucleotidase | Nt5c2 | Q9ES63 | Ubiquitin carboxyl-terminal hydrolase 29 | Usp29 |
| Q61805 | Lipopolysaccharide-binding protein | Lbp | Q8R5J9 | PRA1 family protein 3 | Arl6ip5 | Q3TCX3 | KH homology domain-containing protein 4 | Khdc4 |
| Q6PD03 | Serine/threonine-protein phosphatase 2A 56 kDa regulatory subunit alpha isoform | Ppp2r5a | P07310 | Creatine kinase M-type | Ckm | P51675 | C-C chemokine receptor type 1 | Ccr1 |
| Q9CRC8 | Leucine-rich repeat-containing protein 40 | Lrrc40 | Q60953 | Protein PML | Pml | Q9R0I7 | YLP motif-containing protein 1 | Ylpm1 |
| Q2NL51 | Glycogen synthase kinase-3 alpha | Gsk3a | Q99MQ4 | Asporin | Aspn | Q91ZR1 | Ras-related protein Rab-4B | Rab4b |
| Q91XU0 | ATPase WRNIP1 | Wrnip1 | Q9DCB1 | High mobility group nucleosome-binding domain-containing protein 3 | Hmgn3 | Q7TSI3 | Serine/threonine-protein phosphatase 6 regulatory subunit 1 | Ppp6r1 |
| P35505 | Fumarylacetoacetase | Fah | Q9Z0R4 | Intersectin-1 | Itsn1 | P30355 | Arachidonate 5-lipoxygenase-activating protein | Alox5ap |
| P97470 | Serine/threonine-protein phosphatase 4 catalytic subunit | Ppp4c | Q8VEL2 | Myotubularin-related protein 14 | Mtmr14 | P42230 | Signal transducer and activator of transcription 5A | Stat5a |
| P48193 | Protein 4.1 | Epb41 | Q6ZPJ0 | Testis-expressed protein 2 | Tex2 | Q921U8 | Smoothelin | Smtn |
| P54987 | Cis-aconitate decarboxylase | Acod1 | Q8R502 | Volume-regulated anion channel subunit LRRC8C | Lrrc8c | O35954 | Membrane-associated phosphatidylinositol transfer protein 1 | Pitpnm1 |
| Q9QXZ0 | Microtubule-actin cross-linking factor 1, isoforms 1/2/3/4 | Macf1 | Q64287 | Interferon regulatory factor 4 | Irf4 | Q8K1X1 | WD repeat-containing protein 11 | Wdr11 |
| Q9JL15 | Galectin-8 | Lgals8 | Q99MB1 | Toll-like receptor 3 | Tlr3 | Q9Z0H8 | CAP-Gly domain-containing linker protein 2 | Clip2 |
| Q7TNB8 | Protein strawberry notch homolog 2 | Sbno2 | Q99P30 | Peroxisomal coenzyme A diphosphatase NUDT7 | Nudt7 | Q8BH86 | D-glutamate cyclase, mitochondrial | Dglucy |
| P20664 | DNA primase small subunit | Prim1 | Q9JLM8 | Serine/threonine-protein kinase DCLK1 | Dclk1 | Q8CB87 | Ras-related protein Rab-44 | Rab44 |
| Q921J4 | Ubiquitin-conjugating enzyme E2 S | Ube2s | Q6ZPE2 | Myotubularin-related protein 5 | Sbf1 | Q8K221 | Arfaptin-2 | Arfip2 |
| Q920B9 | FACT complex subunit SPT16 | Supt16h | Q9DC50 | Peroxisomal carnitine O-octanoyltransferase | Crot | Q9Z2L6 | Multiple inositol polyphosphate phosphatase 1 | Minpp1 |
| Q9D0L7 | Armadillo repeat-containing protein 10 | Armc10 | P11214 | Tissue-type plasminogen activator | Plat | Q8BPM2 | Mitogen-activated protein kinase kinase kinase kinase 5 | Map4k5 |
| Q63918 | Caveolae-associated protein 2 | Cavin2 | Q6A026 | Sister chromatid cohesion protein PDS5 homolog A | Pds5a | Q9CZJ6 | Protein Mis18-alpha | Mis18a |
| Q8R121 | Protein Z-dependent protease inhibitor | Serpina10 | Q9CZ96 | Zinc finger CCHC-type and RNA-binding motif-containing protein 1 | Zcrb1 | O88627 | Sodium/nucleoside cotransporter 2 | Slc28a2 |
| P16015 | Carbonic anhydrase 3 | Ca3 | Q3UFS0 | Protein zyg-11 homolog B | Zyg11b | Q3UVG3 | Protein FAM91A1 | Fam91a1 |
| Q8C3J5 | Dedicator of cytokinesis protein 2 | Dock2 | Q8R5L3 | Vam6/Vps39-like protein | Vps39 | Q91YT2 | E3 ubiquitin-protein ligase RNF185 | Rnf185 |
| Q8CEE6 | PAS domain-containing serine/threonine-protein kinase | Pask | Q5NC05 | Transcription termination factor 2 | Ttf2 | Q6NVE8 | WD repeat-containing protein 44 | Wdr44 |
| Q3TJD7 | PDZ and LIM domain protein 7 | Pdlim7 | P08883 | Granzyme F | Gzmf | Q5DW34 | Histone-lysine N-methyltransferase EHMT1 | Ehmt1 |
| Q9CSH3 | Exosome complex exonuclease RRP44 | Dis3 | Q8R307 | Vacuolar protein sorting-associated protein 18 homolog | Vps18 | P97473 | RISC-loading complex subunit TARBP2 | Tarbp2 |
| P83741 | Serine/threonine-protein kinase WNK1 | Wnk1 | Q9JJK5 | Homocysteine-responsive endoplasmic reticulum-resident ubiquitin-like domain member 1 protein | Herpud1 | Q8BPU7 | Engulfment and cell motility protein 1 | Elmo1 |
| P11247 | Myeloperoxidase | Mpo | Q5RL51 | Glutathione S-transferase C-terminal domain-containing protein | Gstcd | P97329 | Kinesin-like protein KIF20A | Kif20a |
| Q61646 | Haptoglobin | Hp | Q6ZQJ5 | DNA replication ATP-dependent helicase/nuclease DNA2 | Dna2 | A2A5R2 | Brefeldin A-inhibited guanine nucleotide-exchange protein 2 | Arfgef2 |
| Q8CFQ3 | RNA helicase aquarius | Aqr | Q8BP00 | IQ calmodulin-binding motif-containing protein 1 | Iqcb1 | Q91Z46 | Dual specificity protein phosphatase 7 | Dusp7 |
| Q06138 | Calcium-binding protein 39 | Cab39 | Q8K3Z9 | Nuclear envelope pore membrane protein POM 121 | Pom121 | Q60604 | Scinderin | Scin |
| Q8JZR0 | Long-chain-fatty-acid--CoA ligase 5 | Acsl5 | Q6NT99 | Dual specificity protein phosphatase 23 | Dusp23 | Q3U182 | CREB-regulated transcription coactivator 2 | Crtc2 |
| Q9QZ73 | DCN1-like protein 1 | Dcun1d1 | Q9EP97 | Sentrin-specific protease 3 | Senp3 | Q9DBT5 | AMP deaminase 2 | Ampd2 |
| O88286 | Protein Wiz | Wiz | Q9R098 | Hepatocyte growth factor activator | Hgfac | Q91X78 | Erlin-1 | Erlin1 |
| Q9WUB3 | Glycogen phosphorylase, muscle form | Pygm | Q64442 | Sorbitol dehydrogenase | Sord | Q3U0M1 | Trafficking protein particle complex subunit 9 | Trappc9 |
| Q9JHS9 | Spliceosome-associated protein CWC15 homolog | Cwc15 | G5E870 | E3 ubiquitin-protein ligase TRIP12 | Trip12 | Q6BCL1 | PML-RARA-regulated adapter molecule 1 | Pram1 |
| Q8WUR0 | Protein C19orf12 homolog | | O88455 | 7-dehydrocholesterol reductase | Dhcr7 | P57746 | V-type proton ATPase subunit D | Atp6v1d |
| E9Q5F9 | Histone-lysine N-methyltransferase SETD2 | Setd2 | Q4QRL3 | Coiled-coil domain-containing protein 88B | Ccdc88b | P97814 | Proline-serine-threonine phosphatase-interacting protein 1 | Pstpip1 |
| Q91YI0 | Argininosuccinate lyase | Asl | O35343 | Importin subunit alpha-3 | Kpna4 | Q9D1J1 | Adaptin ear-binding coat-associated protein 2 | Necap2 |
| Q60960 | Importin subunit alpha-5 | Kpna1 | Q9R1K9 | Centrin-2 | Cetn2 | O08644 | Ephrin type-B receptor 6 | Ephb6 |
| Q5SUA5 | Unconventional myosin-Ig | Myo1g | Q3UFK8 | FERM domain-containing protein 8 | Frmd8 | Q9ES00 | Ubiquitin conjugation factor E4 B | Ube4b |
| Q8BZ36 | RAD50-interacting protein 1 | Rint1 | Q8BWW9 | Serine/threonine-protein kinase N2 | Pkn2 | Q9WTK7 | Serine/threonine-protein kinase STK11 | Stk11 |
| Q8K202 | DNA-directed RNA polymerase I subunit RPA49 | Polr1e | P01642 | Ig kappa chain V-V region L7 (Fragment) | Gm10881 | Q9D2E2 | Target of EGR1 protein 1 | Toe1 |
| A2AWA9 | Rab GTPase-activating protein 1 | Rabgap1 | Q7TNG5 | Echinoderm microtubule-associated protein-like 2 | Eml2 | Q8C5L3 | CCR4-NOT transcription complex subunit 2 | Cnot2 |
| Q9DD18 | D-aminoacyl-tRNA deacylase 1 | Dtd1 | Q9JHI7 | Exosome complex component RRP45 | Exosc9 | Q9D1H7 | Golgi to ER traffic protein 4 homolog | Get4 |
| Q8K0H5 | Transcription initiation factor TFIID subunit 10 | Taf10 | Q99JZ0 | Syntenin-2 | Sdcbp2 | A2ASS6 | Titin | Ttn |
| Q61554 | Fibrillin-1 | Fbn1 | Q9CWR7 | Metalloreductase STEAP1 | Steap1 | Q8BZ03 | Serine/threonine-protein kinase D2 | Prkd2 |
| O35216 | Histone H3-like centromeric protein A | Cenpa | Q99PU7 | Ubiquitin carboxyl-terminal hydrolase BAP1 | Bap1 | Q9CQW2 | ADP-ribosylation factor-like protein 8B | Arl8b |
| P15306 | Thrombomodulin | Thbd | Q9WV03 | Protein FAM50A | Fam50a | Q8CES0 | N-alpha-acetyltransferase 30 | Naa30 |
| Q8CCH2 | NHL repeat-containing protein 3 | Nhlrc3 | Q8BHG2 | CXXC motif containing zinc binding protein | Czib | Q4FZC9 | Nesprin-3 | Syne3 |
| O55057 | Retinal rod rhodopsin-sensitive cGMP 3,5-cyclic phosphodiesterase subunit delta | Pde6d | Q6PB44 | Tyrosine-protein phosphatase non-receptor type 23 | Ptpn23 | P48725 | Pericentrin | Pcnt |
| Q9D8Y1 | Transmembrane protein 126A | Tmem126a | P04918 | Serum amyloid A-3 protein | Saa3 | P50171 | (3R)-3-hydroxyacyl-CoA dehydrogenase | Hsd17b8 |
| Q9EP82 | tRNA (guanine-N(7)-)-methyltransferase non-catalytic subunit WDR4 | Wdr4 | Q9DCF9 | Translocon-associated protein subunit gamma | Ssr3 | Q60809 | CCR4-NOT transcription complex subunit 7 | Cnot7 |
| Q61400 | Carcinoembryonic antigen-related cell adhesion molecule 10 | Ceacam10 | Q91VS7 | Microsomal glutathione S-transferase 1 | Mgst1 | Q8CBE3 | WD repeat-containing protein 37 | Wdr37 |
| Q60963 | Platelet-activating factor acetylhydrolase | Pla2g7 | O70250 | Phosphoglycerate mutase 2 | Pgam2 | Q99KG5 | Lipolysis-stimulated lipoprotein receptor | Lsr |
| Q5DTM8 | E3 ubiquitin-protein ligase BRE1A | Rnf20 | P04247 | Myoglobin | Mb | P20491 | High affinity immunoglobulin epsilon receptor subunit gamma | Fcer1g |
| Q8BHI7 | Elongation of very long chain fatty acids protein 5 | Elovl5 | P11859 | Angiotensinogen | Agt | Q8K0V4 | CCR4-NOT transcription complex subunit 3 | Cnot3 |
| Q91UZ5 | Inositol monophosphatase 2 | Impa2 | Q8BJF9 | Charged multivesicular body protein 2b | Chmp2b | Q80TH2 | Erbin | Erbin |
| P37889 | Fibulin-2 | Fbln2 | Q9CQ89 | Protein CutA | Cuta | Q922J9 | Fatty acyl-CoA reductase 1 | Far1 |
| Q5SX39 | Myosin-4 | Myh4 | P97457 | Myosin regulatory light chain 11 | Myl11 | Q8VC19 | 5-aminolevulinate synthase, non-specific, mitochondrial | Alas1 |
| Q9JM99 | Proteoglycan 4 | Prg4 | P68134 | Actin, alpha skeletal muscle | Acta1 | P70335 | Rho-associated protein kinase 1 | Rock1 |
| Q9JLJ0 | Lipopolysaccharide-induced tumor necrosis factor-alpha factor homolog | Litaf | Q01147 | Cyclic AMP-responsive element-binding protein 1 | Creb1 | A2AQ19 | RNA polymerase-associated protein RTF1 homolog | Rtf1 |
| Q8C6U2 | Solute carrier family 66 member 3 | Slc66a3 | Q8K021 | Secretory carrier-associated membrane protein 1 | Scamp1 |  |  |  |
| Q9ERI2 | Ras-related protein Rab-27A | Rab27a | Q8BI72 | CDKN2A-interacting protein | Cdkn2aip |  |  |  |

**Supplementary Table S7 The information of proteins highly expressed in tumors of the CPAP group.**

| **Protein** | **Description** | **Gene** | **Protein** | **Description** | **Gene** | **Protein** | **Description** | **Gene** |
| --- | --- | --- | --- | --- | --- | --- | --- | --- |
| Q8K2Y7 | Large ribosomal subunit protein uL29m | Mrpl47 | P35492 | Histidine ammonia-lyase | Hal | Q8C5P5 | 5-nucleotidase domain-containing protein 1 | Nt5dc1 |
| Q8VCG3 | WD repeat-containing protein 74 | Wdr74 | Q80X19 | Collagen alpha-1(XIV) chain | Col14a1 | Q6PA06 | Atlastin-2 | Atl2 |
| Q62356 | Follistatin-related protein 1 | Fstl1 | Q9R0Q7 | Prostaglandin E synthase 3 | Ptges3 | Q921H9 | Cytochrome c oxidase assembly factor 7 | Coa7 |
| Q9D883 | Splicing factor U2AF 35 kDa subunit | U2af1 | Q62188 | Dihydropyrimidinase-related protein 3 | Dpysl3 | P62309 | Small nuclear ribonucleoprotein G | Snrpg |
| P05017 | Insulin-like growth factor I | Igf1 | Q8VCZ8 | RNA pseudouridylate synthase domain-containing protein 1 | Rpusd1 | Q9CY34 | NEDD8-conjugating enzyme UBE2F | Ube2f |
| Q3TEW6 | Myelin protein zero-like protein 1 | Mpzl1 | Q9CWR1 | WD repeat-containing protein 73 | Wdr73 | Q60790 | Ras GTPase-activating protein 3 | Rasa3 |
| Q99N84 | Small ribosomal subunit protein mS40 | Mrps18b | P58404 | Striatin-4 | Strn4 | Q9JJN5 | Carboxypeptidase N catalytic chain | Cpn1 |
| Q9JIX0 | Transcription and mRNA export factor ENY2 | Eny2 | Q9CR61 | NADH dehydrogenase [ubiquinone] 1 beta subcomplex subunit 7 | Ndufb7 | P61967 | AP-1 complex subunit sigma-1A | Ap1s1 |
| Q9JHI5 | Isovaleryl-CoA dehydrogenase, mitochondrial | Ivd | P01867 | Immunoglobulin heavy constant gamma 2B | Ighg2b | Q9BDB7 | Interferon-induced protein 44-like | Ifi44l |
| P03987 | Ig gamma-3 chain C region |  | P01630 | Ig kappa chain V-II region 7S34.1 |  | Q810U5 | Coiled-coil domain-containing protein 50 | Ccdc50 |
| P43024 | Cytochrome c oxidase subunit 6A1, mitochondrial | Cox6a1 | P15626 | Glutathione S-transferase Mu 2 | Gstm2 | Q80Y98 | Phospholipase DDHD2 | Ddhd2 |
| P62874 | Guanine nucleotide-binding protein G(I)/G(S)/G(T) subunit beta-1 | Gnb1 | P01592 | Immunoglobulin J chain | Jchain | Q91VZ6 | Stromal membrane-associated protein 1 | Smap1 |
| Q8K339 | DNA/RNA-binding protein KIN17 | Kin | P01746 | Ig heavy chain V region 93G7 |  | Q9DBU0 | Transmembrane 9 superfamily member 1 | Tm9sf1 |
| Q9D0T1 | NHP2-like protein 1 | Snu13 | Q61733 | Small ribosomal subunit protein mS31 | Mrps31 | P42209 | Septin-1 | Septin1 |
| O88396 | GrpE protein homolog 2, mitochondrial | Grpel2 | Q9R0M4 | Podocalyxin | Podxl | Q8C0E3 | E3 ubiquitin-protein ligase TRIM47 | Trim47 |
| P97314 | Cysteine and glycine-rich protein 2 | Csrp2 | P08074 | Carbonyl reductase [NADPH] 2 | Cbr2 | O88643 | Serine/threonine-protein kinase PAK 1 | Pak1 |
| Q9CR98 | Protein FAM136A | Fam136a | P01878 | Ig alpha chain C region |  | Q9R0L6 | Pericentriolar material 1 protein | Pcm1 |
| P12023 | Amyloid-beta precursor protein | App | Q8CGE8 | Interferon-activable protein 205-A | Ifi205a | Q9DB43 | Zinc finger protein-like 1 | Zfpl1 |
| Q91XB0 | Three-prime repair exonuclease 1 | Trex1 | Q99JY3 | GTPase IMAP family member 4 | Gimap4 | Q6R891 | Neurabin-2 | Ppp1r9b |
| P0CW02 | Lymphocyte antigen 6C1 | Ly6c1 | P03893 | NADH-ubiquinone oxidoreductase chain 2 | mt-Nd2 | Q9Z2A9 | Glutathione hydrolase 5 proenzyme | Ggt5 |
| Q9CQX2 | Cytochrome b5 type B | Cyb5b | P28798 | Progranulin | Grn | P41731 | CD63 antigen | Cd63 |
| Q9CZL5 | Pterin-4-alpha-carbinolamine dehydratase 2 | Pcbd2 | Q8BHL8 | Proteasome inhibitor PI31 subunit | Psmf1 | Q60823 | RAC-beta serine/threonine-protein kinase | Akt2 |
| P70289 | Receptor-type tyrosine-protein phosphatase V | Ptprv | P01807 | Ig heavy chain V region X44 |  | Q9D937 | Uncharacterized protein C11orf98 homolog |  |
| Q9D3P8 | Plasminogen receptor (KT) | Plgrkt | Q8JZK9 | Hydroxymethylglutaryl-CoA synthase, cytoplasmic | Hmgcs1 | Q9CPR8 | Non-structural maintenance of chromosomes element 3 homolog | Nsmce3 |
| Q8BJU9 | Peptide chain release factor 1-like, mitochondrial | Mtrf1l | Q8JZL7 | Ras-GEF domain-containing family member 1B | Rasgef1b | P26323 | Friend leukemia integration 1 transcription factor | Fli1 |
| Q9CQS2 | H/ACA ribonucleoprotein complex subunit 3 | Nop10 | Q9CQJ8 | NADH dehydrogenase [ubiquinone] 1 beta subcomplex subunit 9 | Ndufb9 | A2AHC3 | Calmodulin-regulated spectrin-associated protein 1 | Camsap1 |
| Q91VJ2 | Caveolae-associated protein 3 | Cavin3 | P01750 | Ig heavy chain V region 102 |  | Q8BHL3 | TBC1 domain family member 10B | Tbc1d10b |
| Q80YV2 | Zinc finger C3HC-type protein 1 | Zc3hc1 | P25785 | Metalloproteinase inhibitor 2 | Timp2 | Q8C0L6 | Peroxisomal N(1)-acetyl-spermine/spermidine oxidase | Paox |
| Q9Z121 | C-C motif chemokine 8 | Ccl8 | Q9D172 | Glutamine amidotransferase-like class 1 domain-containing protein 3, mitochondrial | Gatd3 | Q8BU11 | TOX high mobility group box family member 4 | Tox4 |
| Q8CBB9 | S-adenosylmethionine-dependent nucleotide dehydratase RSAD2 | Rsad2 | P01741 | Ig heavy chain V region |  | Q8K2F8 | Protein LSM14 homolog A | Lsm14a |
| Q8R0F3 | Formylglycine-generating enzyme | Sumf1 | Q99K28 | ADP-ribosylation factor GTPase-activating protein 2 | Arfgap2 | Q9DBB8 | Trans-1,2-dihydrobenzene-1,2-diol dehydrogenase | Dhdh |
| Q9JJI8 | Large ribosomal subunit protein eL38 | Rpl38 | O55242 | Sigma non-opioid intracellular receptor 1 | Sigmar1 | O54804 | Choline kinase alpha | Chka |
| P62892 | Large ribosomal subunit protein eL39 | Rpl39 | P18527 | Ig heavy chain V region 914 |  | P01657 | Ig kappa chain V-III region PC 2413 |  |
| O35943 | Frataxin, mitochondrial | Fxn | P51175 | Protoporphyrinogen oxidase | Ppox | A6H5X4 | PHD finger protein 11 | Phf11 |
| P18524 | Ig heavy chain V region RF |  | Q9WTL7 | Acyl-protein thioesterase 2 | Lypla2 | P62915 | Transcription initiation factor IIB | Gtf2b |
| P01638 | Ig kappa chain V-V region L6 (Fragment) |  | Q9DCB8 | Iron-sulfur cluster assembly 2 homolog, mitochondrial | Isca2 | Q8K1E0 | Syntaxin-5 | Stx5 |
| Q8BMC4 | Nucleolar protein 9 | Nop9 | Q9R099 | Transducin beta-like protein 2 | Tbl2 | Q03160 | Growth factor receptor-bound protein 7 | Grb7 |
| Q01339 | Beta-2-glycoprotein 1 | Apoh | P07214 | SPARC | Sparc | Q8R310 | Transmembrane and coiled-coil domain protein 3 | Tmcc3 |
| Q8K182 | Complement component C8 alpha chain | C8a | Q99LY9 | NADH dehydrogenase [ubiquinone] iron-sulfur protein 5 | Ndufs5 | Q8R1F9 | Ribonuclease P protein subunit p40 | Rpp40 |
| O88574 | Histone deacetylase complex subunit SAP30 | Sap30 | Q99JX3 | Golgi reassembly-stacking protein 2 | Gorasp2 | Q8BHN1 | Gamma-taxilin | Txlng |
| Q80TI1 | Pleckstrin homology domain-containing family H member 1 | Plekhh1 | Q9CPZ8 | COX assembly mitochondrial protein homolog | Cmc1 | Q62176 | RNA-binding protein 38 | Rbm38 |
| Q91VJ5 | Polyglutamine-binding protein 1 | Pqbp1 | P01672 | Ig kappa chain V-III region PC 7940 |  | Q9DCL2 | Cytosolic iron-sulfur assembly component 2A | Ciao2a |
| P83882 | Large ribosomal subunit protein eL42 | Rpl36a | Q80XC2 | tRNA (adenine(58)-N(1))-methyltransferase catalytic subunit TRMT61A | Trmt61a | Q9CQZ5 | NADH dehydrogenase [ubiquinone] 1 alpha subcomplex subunit 6 | Ndufa6 |
| O70546 | Lysine-specific demethylase 6A | Kdm6a | P14430 | H-2 class I histocompatibility antigen, Q8 alpha chain | H2-Q8 | O54782 | Epididymis-specific alpha-mannosidase | Man2b2 |
| P01837 | Immunoglobulin kappa constant | Igkc | Q8BX09 | Retinoblastoma-binding protein 5 | Rbbp5 | Q9D281 | Protein Noxp20 | Fam114a1 |
| Q99LC5 | Electron transfer flavoprotein subunit alpha, mitochondrial | Etfa | Q8BVE8 | Histone-lysine N-methyltransferase NSD2 | Nsd2 | P97808 | FXYD domain-containing ion transport regulator 5 | Fxyd5 |
| Q8CGU1 | Calcium-binding and coiled-coil domain-containing protein 1 | Calcoco1 | Q9CR46 | Spindle and kinetochore-associated protein 2 | Ska2 | D3YZP9 | Coiled-coil domain-containing protein 6 | Ccdc6 |
| P33611 | DNA polymerase alpha subunit B | Pola2 | P14427 | H-2 class I histocompatibility antigen, D-P alpha chain | H2-D1 | Q80U87 | Ubiquitin carboxyl-terminal hydrolase 8 | Usp8 |
| Q9JHS4 | ATP-dependent Clp protease ATP-binding subunit clpX-like, mitochondrial | Clpx | O70423 | Membrane primary amine oxidase | Aoc3 | Q9Z0H7 | B-cell lymphoma/leukemia 10 | Bcl10 |
| Q9D0G0 | Large ribosomal subunit protein mL65 | Mrps30 | Q08857 | Platelet glycoprotein 4 | Cd36 | Q8BXA5 | Lipid scramblase CLPTM1L | Clptm1l |
| Q8C4V1 | Rho GTPase-activating protein 24 | Arhgap24 | Q99N90 | Large ribosomal subunit protein bL36m | Mrpl36 | Q9JMA2 | Queuine tRNA-ribosyltransferase catalytic subunit 1 | Qtrt1 |
| Q60994 | Adiponectin | Adipoq | P26262 | Plasma kallikrein | Klkb1 | Q8C1S0 | Mediator of RNA polymerase II transcription subunit 19 | Med19 |
| Q05920 | Pyruvate carboxylase, mitochondrial | Pc | P01865 | Ig gamma-2A chain C region, membrane-bound form | Igh-1a | Q9WVD5 | Mitochondrial ornithine transporter 1 | Slc25a15 |
| Q64471 | Glutathione S-transferase theta-1 | Gstt1 | P01844 | Ig lambda-2 chain C region | Iglc2 | Q6NXN1 | SUZ domain-containing protein 1 | Szrd1 |
| Q922E6 | FAST kinase domain-containing protein 2, mitochondrial | Fastkd2 | Q8VI63 | MOB kinase activator 2 | Mob2 | Q3UQU0 | Bromodomain-containing protein 9 | Brd9 |
| O89017 | Legumain | Lgmn | Q61510 | E3 ubiquitin/ISG15 ligase TRIM25 | Trim25 | O88697 | Serine/threonine-protein kinase 16 | Stk16 |
| Q9Z1J3 | Cysteine desulfurase | Nfs1 | Q07797 | Galectin-3-binding protein | Lgals3bp | Q8K3G9 | DCC-interacting protein 13-beta | Appl2 |
| Q8C5Q4 | G-rich sequence factor 1 | Grsf1 | Q64282 | Interferon-induced protein with tetratricopeptide repeats 1 | Ifit1 | Q91VX2 | Ubiquitin-associated protein 2 | Ubap2 |
| Q62165 | Dystroglycan 1 | Dag1 | Q9JIB0 | Ran guanine nucleotide release factor | Rangrf | Q9D7A6 | Signal recognition particle 19 kDa protein | Srp19 |
| P07724 | Albumin | Alb | P70429 | Ena/VASP-like protein | Evl | Q3TWL2 | Type 1 phosphatidylinositol 4,5-bisphosphate 4-phosphatase | Pip4p1 |
| Q9CSN1 | SNW domain-containing protein 1 | Snw1 | P01869 | Ig gamma-1 chain C region, membrane-bound form | Ighg1 | Q8C1B1 | Calmodulin-regulated spectrin-associated protein 2 | Camsap2 |
| P21958 | Antigen peptide transporter 1 | Tap1 | Q14B71 | Cell division cycle-associated protein 2 | Cdca2 | P63213 | Guanine nucleotide-binding protein G(I)/G(S)/G(O) subunit gamma-2 | Gng2 |
| Q8BW00 | Probable peptidyl-tRNA hydrolase | Ptrh1 | Q9ES89 | Exostosin-like 2 | Extl2 | P04184 | Thymidine kinase, cytosolic | Tk1 |
| Q4FZD7 | Inactive serine/threonine-protein kinase PLK5 | Plk5 | Q9Z0H3 | SWI/SNF-related matrix-associated actin-dependent regulator of chromatin subfamily B member 1 | Smarcb1 | O88746 | Target of Myb1 membrane trafficking protein | Tom1 |
| Q99NB8 | Ubiquilin-4 | Ubqln4 | Q8BH97 | Reticulocalbin-3 | Rcn3 | Q3TN34 | MICAL-like protein 2 | Micall2 |
| Q52KR2 | Leucine-rich repeats and immunoglobulin-like domains protein 2 | Lrig2 | Q69Z37 | Sterile alpha motif domain-containing protein 9-like | Samd9l | Q9D6T0 | Nitric oxide synthase-interacting protein | Nosip |
| Q9D855 | Cytochrome b-c1 complex subunit 7 | Uqcrb | Q61730 | Interleukin-1 receptor accessory protein | Il1rap | P03899 | NADH-ubiquinone oxidoreductase chain 3 | mt-Nd3 |
| Q63959 | Potassium voltage-gated channel subfamily C member 3 | Kcnc3 | A0A0G2JDV3 | Guanylate-binding protein 6 | Gbp6 | Q8BH93 | MAPK-interacting and spindle-stabilizing protein-like | Mapk1ip1l |
| Q9QWH1 | Polyhomeotic-like protein 2 | Phc2 | Q8VBT6 | Apolipoprotein B receptor | Apobr | Q9CQE7 | Endoplasmic reticulum-Golgi intermediate compartment protein 3 | Ergic3 |
| Q9QYI3 | DnaJ homolog subfamily C member 7 | Dnajc7 | P49443 | Protein phosphatase 1A | Ppm1a | Q99MD6 | Thioredoxin reductase 3 | Txnrd3 |
| Q6A068 | Cell division cycle 5-like protein | Cdc5l | O35601 | FYN-binding protein 1 | Fyb1 | P01787 | Ig heavy chain V regions TEPC 15/S107/HPCM1/HPCM2/HPCM3 |  |
| Q9QWK4 | CD5 antigen-like | Cd5l | P07309 | Transthyretin | Ttr | Q6P3B9 | Putative ribosome-binding factor A, mitochondrial | Rbfa |
| Q8VCL2 | Protein SCO2 homolog, mitochondrial | Sco2 | P01801 | Ig heavy chain V-III region J606 |  | O89100 | GRB2-related adaptor protein 2 | Grap2 |
| Q99M04 | Lipoyl synthase, mitochondrial | Lias | P42703 | Leukemia inhibitory factor receptor | Lifr | Q8BP71 | RNA binding protein fox-1 homolog 2 | Rbfox2 |
| Q64311 | Protein N-terminal asparagine amidohydrolase | Ntan1 | O89086 | RNA-binding protein 3 | Rbm3 | Q8BMS4 | Ubiquinone biosynthesis O-methyltransferase, mitochondrial | Coq3 |
| Q8K354 | Carbonyl reductase [NADPH] 3 | Cbr3 | P84750 | Ig kappa chain V region Mem5 (Fragment) |  | Q8R3P6 | Integrator complex subunit 14 | Ints14 |
| P06330 | Ig heavy chain V region AC38 205.12 |  | Q9QY24 | Z-DNA-binding protein 1 | Zbp1 | Q8CG19 | Latent-transforming growth factor beta-binding protein 1 | Ltbp1 |
| Q00731 | Vascular endothelial growth factor A, long form | Vegfa | Q8VEA4 | Mitochondrial intermembrane space import and assembly protein 40 | Chchd4 | Q8K1I3 | Secreted phosphoprotein 24 | Spp2 |
| P48024 | Eukaryotic translation initiation factor 1 | Eif1 | Q8K5B2 | Multiple coagulation factor deficiency protein 2 homolog | Mcfd2 | O55125 | Protein NipSnap homolog 1 | Nipsnap1 |
| Q8CAK1 | Putative transferase CAF17 homolog, mitochondrial | Iba57 | Q6PD26 | GPI transamidase component PIG-S | Pigs | Q9EQN3 | TSC22 domain family protein 4 | Tsc22d4 |
| Q9DD06 | Retinoic acid receptor responder protein 2 | Rarres2 | Q06770 | Corticosteroid-binding globulin | Serpina6 | Q9CR13 | Protein FMC1 homolog | Fmc1 |
| Q9D7S7 | Large ribosomal subunit protein eL22-like 1 | Rpl22l1 | Q9R002 | Interferon-activable protein 202 | Ifi202 | P33434 | 72 kDa type IV collagenase | Mmp2 |
| P18528 | Ig heavy chain V region 6.96 |  | Q80SW1 | S-adenosylhomocysteine hydrolase-like protein 1 | Ahcyl1 | P29594 | Caspase-2 | Casp2 |
| P18531 | Ig heavy chain V region 3-6 | Ighv3-6 | Q8BSL7 | ADP-ribosylation factor 2 | Arf2 | Q5I012 | Putative sodium-coupled neutral amino acid transporter 10 | Slc38a10 |
| O54962 | Barrier-to-autointegration factor | Banf1 | Q64112 | Interferon-induced protein with tetratricopeptide repeats 2 | Ifit2 | P01821 | Ig heavy chain V region MC101 |  |
| Q9QZH3 | Peptidyl-prolyl cis-trans isomerase E | Ppie | Q9JKB3 | Y-box-binding protein 3 | Ybx3 | Q91ZF0 | DnaJ homolog subfamily C member 24 | Dnajc24 |
| Q9DCJ9 | N-acetylneuraminate lyase | Npl | Q8BHJ5 | F-box-like/WD repeat-containing protein TBL1XR1 | Tbl1xr1 | Q6PDX6 | E3 ubiquitin-protein ligase Rnf220 | Rnf220 |
| Q9DB60 | Prostamide/prostaglandin F synthase | Prxl2b | Q8CAS9 | Protein mono-ADP-ribosyltransferase PARP9 | Parp9 | P56392 | Cytochrome c oxidase subunit 7A1, mitochondrial | Cox7a1 |
| O88384 | Vesicle transport through interaction with t-SNAREs homolog 1B | Vti1b | Q3UQ44 | Ras GTPase-activating-like protein IQGAP2 | Iqgap2 | P62500 | TSC22 domain family protein 1 | Tsc22d1 |
| P11680 | Properdin | Cfp | Q91YR9 | Prostaglandin reductase 1 | Ptgr1 | Q9CX48 | Zinc finger CCHC domain-containing protein 10 | Zcchc10 |
| Q99KN2 | Probable cytosolic iron-sulfur protein assembly protein CIAO1 | Ciao1 | Q99JT2 | Serine/threonine-protein kinase 26 | Stk26 | Q80X32 | UPF0461 protein C5orf24 homolog |  |
| O88532 | Zinc finger RNA-binding protein | Zfr | P39654 | Polyunsaturated fatty acid lipoxygenase ALOX15 | Alox15 | Q9D924 | Iron-sulfur cluster assembly 1 homolog, mitochondrial | Isca1 |
| P01872 | Immunoglobulin heavy constant mu | Ighm | Q9DCE9 | Immunity-related GTPase family M protein 3 | Igtp | Q9WUP4 | Polyprenol reductase | Srd5a3 |
| Q9CX80 | Cytoglobin | Cygb | Q925E7 | Serine/threonine-protein phosphatase 2A 55 kDa regulatory subunit B delta isoform | Ppp2r2d | Q921N8 | G protein pathway suppressor 2 | Gps2 |
| P43025 | Tetranectin | Clec3b | Q7TSC1 | Protein PRRC2A | Prrc2a | O88597 | Beclin-1 | Becn1 |
| Q00724 | Retinol-binding protein 4 | Rbp4 | P58871 | 182 kDa tankyrase-1-binding protein | Tnks1bp1 | Q9D710 | Thioredoxin-related transmembrane protein 2 | Tmx2 |
| Q9CWX4 | Pseudouridylate synthase RPUSD4, mitochondrial | Rpusd4 | Q8R5F7 | Interferon-induced helicase C domain-containing protein 1 | Ifih1 | Q9CXK9 | RNA-binding protein 33 | Rbm33 |
| Q8BH57 | WD repeat-containing protein 48 | Wdr48 | Q8VDQ8 | NAD-dependent protein deacetylase sirtuin-2 | Sirt2 | Q3TRM4 | Patatin-like phospholipase domain-containing protein 6 | Pnpla6 |
| P35951 | Low-density lipoprotein receptor | Ldlr | Q8VHK9 | ATP-dependent DNA/RNA helicase DHX36 | Dhx36 | Q9CR70 | EKC/KEOPS complex subunit Lage3 | Lage3 |
| Q8BYJ6 | TBC1 domain family member 4 | Tbc1d4 | P01900 | H-2 class I histocompatibility antigen, D-D alpha chain | H2-D1 | Q00558 | 40-kDa huntingtin-associated protein | F8a1 |
| Q9JJG9 | Nitric oxide-associated protein 1 | Noa1 | Q8BV49 | Pyrin and HIN domain-containing protein 1 | Pyhin1 | Q9JJ00 | Phospholipid scramblase 1 | Plscr1 |
| Q9CRB2 | H/ACA ribonucleoprotein complex subunit 2 | Nhp2 | Q9WVL2 | Signal transducer and activator of transcription 2 | Stat2 | Q6GU68 | Immunoglobulin superfamily containing leucine-rich repeat protein | Islr |
| P26928 | Hepatocyte growth factor-like protein | Mst1 | Q5SFM8 | RNA-binding protein 27 | Rbm27 | P25425 | POU domain, class 2, transcription factor 1 | Pou2f1 |
| O89020 | Afamin | Afm | P97927 | Laminin subunit alpha-4 | Lama4 | Q8BUV8 | Protein GPR107 | Gpr107 |
| P02468 | Laminin subunit gamma-1 | Lamc1 | P52196 | Thiosulfate sulfurtransferase | Tst | Q8K2Q7 | BRO1 domain-containing protein BROX | Brox |
| Q9D773 | Large ribosomal subunit protein uL2m | Mrpl2 | Q61292 | Laminin subunit beta-2 | Lamb2 | Q8CE50 | Sorting nexin-30 | Snx30 |
| P12388 | Plasminogen activator inhibitor 2, macrophage | Serpinb2 | Q9D7S9 | Charged multivesicular body protein 5 | Chmp5 | Q8R1L4 | ER lumen protein-retaining receptor 3 | Kdelr3 |
| Q9JKA5 | Cell surface A33 antigen | Gpa33 | Q9D7E3 | Esterase OVCA2 | Ovca2 | P26231 | Catenin alpha-1 | Ctnna1 |
| G5E897 | Protein O-glucosyltransferase 3 | Poglut3 | Q8BY35 | FYVE, RhoGEF and PH domain-containing protein 2 | Fgd2 | Q9D0I4 | Syntaxin-17 | Stx17 |
| Q9CQP0 | Large ribosomal subunit protein bL33m | Mrpl33 | P47809 | Dual specificity mitogen-activated protein kinase kinase 4 | Map2k4 | Q8K1N2 | Pleckstrin homology-like domain family B member 2 | Phldb2 |
| Q8K199 | COX assembly mitochondrial protein 2 homolog | Cmc2 | Q8K482 | EMILIN-2 | Emilin2 | P07743 | BPI fold-containing family A member 2 | Bpifa2 |
| P01644 | Ig kappa chain V-V region HP R16.7 |  | Q8VCH8 | UBX domain-containing protein 4 | Ubxn4 | Q8C167 | Prolyl endopeptidase-like | Prepl |
| P03976 | Ig kappa chain V-II region 17S29.1 |  | Q8CH25 | SAFB-like transcription modulator | Sltm | Q8VI33 | Transcription initiation factor TFIID subunit 9 | Taf9 |
| Q9CRB6 | Tubulin polymerization-promoting protein family member 3 | Tppp3 | Q8BHG9 | CGG triplet repeat-binding protein 1 | Cggbp1 | Q9D3B1 | Very-long-chain (3R)-3-hydroxyacyl-CoA dehydratase 2 | Hacd2 |
| Q80YC5 | Coagulation factor XII | F12 | Q6NWW9 | Fibronectin type III domain-containing protein 3B | Fndc3b | Q8BJ64 | Choline dehydrogenase, mitochondrial | Chdh |
| Q8K273 | ER membrane protein complex subunit 5 | Mmgt1 | Q91XC8 | Death-associated protein 1 | Dap | Q99N20 | Breast cancer metastasis-suppressor 1 homolog | Brms1 |
| Q91VN4 | MICOS complex subunit Mic25 | Chchd6 | Q9EPQ7 | StAR-related lipid transfer protein 5 | Stard5 | O35566 | CD151 antigen | Cd151 |
| P01786 | Ig heavy chain V region MOPC 47A |  | Q9Z2Z9 | Glutamine--fructose-6-phosphate aminotransferase [isomerizing] 2 | Gfpt2 | Q921Y4 | Molybdate-anion transporter | Mfsd5 |
| P09813 | Apolipoprotein A-II | Apoa2 | Q3TFD2 | Lysophosphatidylcholine acyltransferase 1 | Lpcat1 | Q8CH40 | Nucleoside diphosphate-linked moiety X motif 6 | Nudt6 |
| Q62241 | U1 small nuclear ribonucleoprotein C | Snrpc | Q8VCF0 | Mitochondrial antiviral-signaling protein | Mavs | Q9CQT5 | Proteasome maturation protein | Pomp |
| Q9D2P4 | Ubiquitin-related modifier 1 | Urm1 | O08912 | Polypeptide N-acetylgalactosaminyltransferase 1 | Galnt1 | Q4VC33 | E3 ubiquitin-protein transferase MAEA | Maea |
| P01631 | Ig kappa chain V-II region 26-10 |  | P62965 | Cellular retinoic acid-binding protein 1 | Crabp1 | Q3V1M1 | Immunoglobulin superfamily member 10 | Igsf10 |
| Q9D7Z3 | Nucleolar protein 7 | Nol7 | Q8CGN5 | Perilipin-1 | Plin1 | Q8R2Y9 | SOSS complex subunit B1 | Nabp2 |
| Q9QWF0 | Chromatin assembly factor 1 subunit A | Chaf1a | Q7TNG8 | Probable D-lactate dehydrogenase, mitochondrial | Ldhd | Q99NA9 | Polycomb group RING finger protein 6 | Pcgf6 |
| Q921W4 | Quinone oxidoreductase-like protein 1 | Cryzl1 | Q8R1J9 | Torsin-2A | Tor2a | Q9DC04 | Regulator of G-protein signaling 3 | Rgs3 |
| Q9D5N8 | Spermidine/spermine N(1)-acetyltransferase-like protein 1 | Satl1 | Q3TB48 | Transmembrane protein 104 | Tmem104 | Q9QZ82 | Cholesterol side-chain cleavage enzyme, mitochondrial | Cyp11a1 |
| Q7TMX5 | Protein SHQ1 homolog | Shq1 | L7N1X6 | F-box/WD repeat-containing protein 15 | Fbxw15 |  |  |  |
